# Progranulin signaling in sepsis, community-acquired bacterial pneumonia and COVID-19: a comparative, observational study

**DOI:** 10.1186/s40635-021-00406-7

**Published:** 2021-09-03

**Authors:** Florian Brandes, Melanie Borrmann, Dominik Buschmann, Agnes S. Meidert, Marlene Reithmair, Markus Langkamp, Lutz Pridzun, Benedikt Kirchner, Jean-Noël Billaud, Nirav M. Amin, Joseph C. Pearson, Matthias Klein, Daniela Hauer, Clarissa Gevargez Zoubalan, Anja Lindemann, Alexander Choukér, Thomas W. Felbinger, Ortrud K. Steinlein, Michael W. Pfaffl, Ines Kaufmann, Gustav Schelling

**Affiliations:** 1 Department of Anaesthesiology, University Hospital, Ludwig-Maximilians-University of Munich, Munich, Germany; 2 Division of Animal Physiology and Immunology, TUM School of Life Sciences Weihenstephan, Technical University of Munich, Munich, Germany; 3 Institute of Human Genetics, University Hospital, Ludwig-Maximilians-University, Munich, Germany; 4 MEDIAGNOST Company, Aspenhausstr. 25, 72770 Reutlingen, Germany; 5 QIAGEN Digital Insights, Redwood City, USA; 6 Department of Neurology, University Hospital, Ludwig-Maximilians-University of Munich, Munich, Germany; 7 Department of Anaesthesiology, Neuperlach Hospital, City Hospitals of Munich, Munich, Germany

**Keywords:** Sepsis, Pneumonia, COVID-19, Progranulin, Procalcitonin, Sensitivity, Specificity, Gene networks

## Abstract

**Background:**

Progranulin is a widely expressed pleiotropic growth factor with a central regulatory effect during the early immune response in sepsis. Progranulin signaling has not been systematically studied and compared between sepsis, community-acquired pneumonia (CAP), COVID-19 pneumonia and a sterile systemic inflammatory response (SIRS). We delineated molecular networks of progranulin signaling by next-generation sequencing (NGS), determined progranulin plasma concentrations and quantified the diagnostic performance of progranulin to differentiate between the above-mentioned disorders using the established biomarkers procalcitonin (PCT), interleukin-6 (IL-6) and C-reactive protein (CRP) for comparison.

**Methods:**

The diagnostic performance of progranulin was operationalized by calculating AUC and ROC statistics for progranulin and established biomarkers in 241 patients with sepsis, 182 patients with SIRS, 53 patients with CAP, 22 patients with COVID-19 pneumonia and 53 healthy volunteers. miRNAs and mRNAs in blood cells from sepsis patients (*n* = 7) were characterized by NGS and validated by RT-qPCR in an independent cohort (*n* = 39) to identify canonical gene networks associated with upregulated progranulin at sepsis onset.

**Results:**

Plasma concentrations of progranulin (ELISA) in patients with sepsis were 57.5 (42.8–84.9, Q25–Q75) ng/ml and significantly higher than in CAP (38.0, 33.5–41.0 ng/ml, *p* < 0.001), SIRS (29.0, 25.0–35.0 ng/ml, *p* < 0.001) and the healthy state (28.7, 25.5–31.7 ng/ml, *p* < 0.001). Patients with COVID-19 had significantly higher progranulin concentrations than patients with CAP (67.6, 56.6–96.0 vs. 38.0, 33.5–41.0 ng/ml, *p* < 0.001). The diagnostic performance of progranulin for the differentiation between sepsis vs. SIRS (*n* = 423) was comparable to that of procalcitonin. AUC was 0.90 (95% CI = 0.87–0.93) for progranulin and 0.92 (CI = 0.88–0.96, *p* = 0.323) for procalcitonin. Progranulin showed high discriminative power to differentiate bacterial CAP from COVID-19 (sensitivity 0.91, specificity 0.94, AUC 0.91 (CI = 0.8–1.0) and performed significantly better than PCT, IL-6 and CRP. NGS and partial RT-qPCR confirmation revealed a transcriptomic network of immune cells with upregulated progranulin and sortilin transcripts as well as toll-like-receptor 4 and tumor-protein 53, regulated by miR-16 and others.

**Conclusions:**

Progranulin signaling is elevated during the early antimicrobial response in sepsis and differs significantly between sepsis, CAP, COVID-19 and SIRS. This suggests that progranulin may serve as a novel indicator for the differentiation between these disorders.

*Trial registration*: Clinicaltrials.gov registration number NCT03280576 Registered November 19, 2015.

**Supplementary Information:**

The online version contains supplementary material available at 10.1186/s40635-021-00406-7.

## Background

Approximately 30 million cases of sepsis associated with more than 6 million annual deaths occur worldwide [1]. Early disease detection and prompt initiation of adequate therapy are regarded as key steps to improve the overall outcome of sepsis [2]. Pneumonia represents the fourth leading global cause of deaths and the deadliest communicable disease, causative for three million deaths worldwide [3]. With the current SARS-CoV-2 pandemic, this fact has very recently been brought to worldwide attention.

The early clinical signs of sepsis and lower respiratory tract infections are unreliable and often difficult to differentiate from the noninfectious SIRS. The inappropriate use of broad-spectrum antibiotics in SIRS or viral pneumonia results in increased antibiotic resistance and costs [4], and also delays adequate treatment of the underlying non-bacterial disorder.

Rapidly available biomarkers with high sensitivity and specificity are required to detect and to differentiate these disorders in a timely fashion. Presently, available markers such as procalcitonin, C-reactive protein or cytokines (in particular interleukin-6) have proinflammatory effects, are closely related to the presence of inflammation per se and have limited sensitivity and specificity to establish the diagnosis of pneumonia and sepsis and in order to reduce mortality [5, 6].

Progranulin is a widely expressed pleiotropic growth factor with multiple roles in neurodegeneration [7] and cancer [8] as well as inflammation and immunity [9]. Progranulin also has a central function in the regulation of the host response during the early stages of infection [10]. In contrast to commonly used biomarkers, progranulin is known to exert anti-inflammatory effects by increasing the production of interleukin-10 [11], thus reducing signaling through TNF1 [12] and TLR4 [13]. The central regulatory effects of progranulin in the immune response and the fact that increased progranulin plasma concentrations have been described in small observational studies in patients with sepsis [10] and bacterial pneumonia [14] suggest that this compound could serve as a future biomarker for these disorders.

We investigated the relationship between progranulin plasma levels and disease severity in patients with sepsis, community-acquired pneumonia including COVID-19 and delineated the biologic function of progranulin by molecular network analysis in order to demonstrate the logical validity of using this protein as a possible biomarker. We then compared the performance characteristic of progranulin as a biologic marker for the differentiation between sepsis, the systemic inflammatory response to major surgery, bacterial pneumonia and COVID-19 to that of the established indicators procalcitonin, interleukin-6 and C-reactive protein.

## Methods

### Study groups

**Fig. 1:**
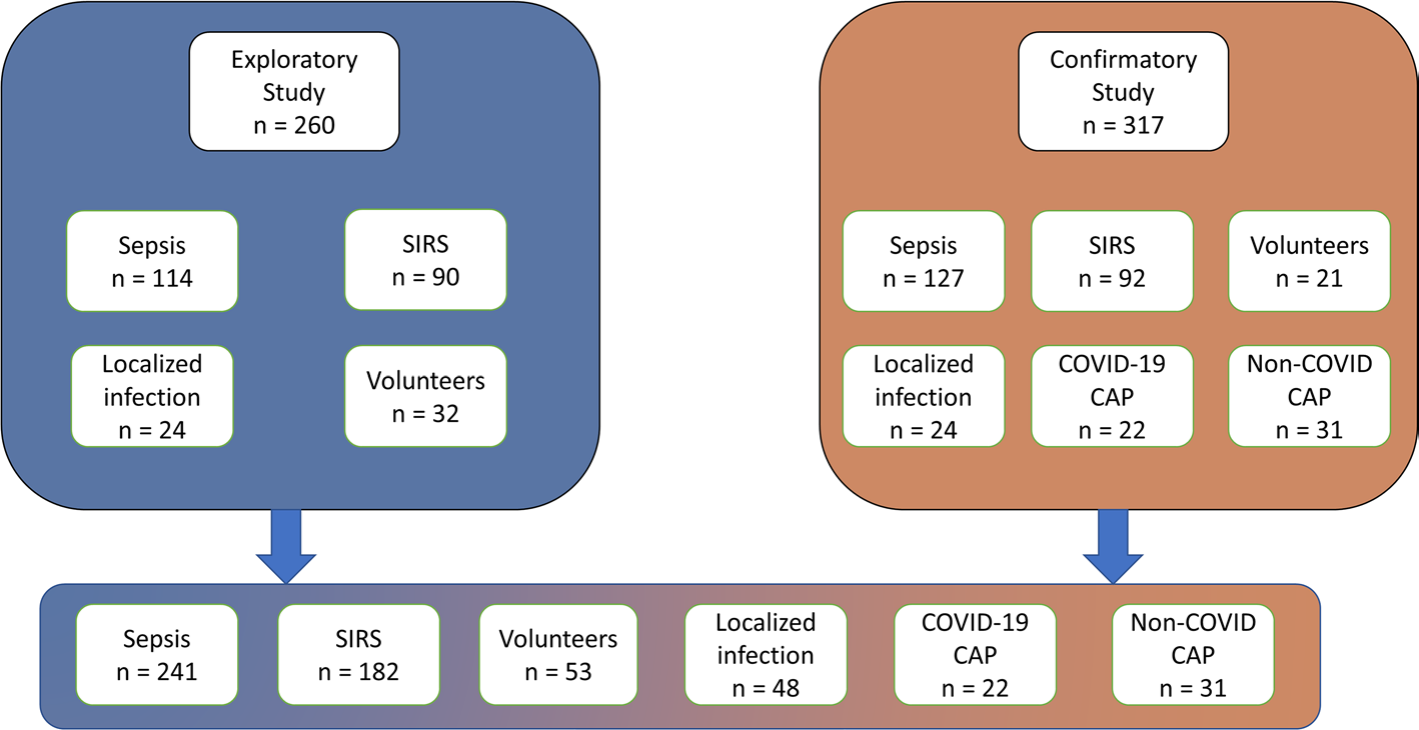
Flowchart describing the origin of the study population, divided by exploratory and confirmatory study. Overall, the exploratory study population consists of 260 individuals (including 114 patients with sepsis, 90 patients with SIRS, 24 patients with localized infection and 32 volunteers) and the confirmatory sample consists of 317 individuals (including 127 patients with sepsis, 92 patients with SIRS, 24 patients with localized infection, 22 patients with a COVID-19 CAP, 31 patients with non-COVID-19 CAP as well as 21 volunteers). The community-acquired pneumonia (COVID and non-COVID) groups are only represented in the confirmatory group

We prospectively studied 577 individuals recruited from two large medical centers in the Munich area. The study sample included 241 patients with sepsis; 114 sepsis patients were investigated as an initial exploratory sample and 127 additional patients served as an independent confirmatory sample that was recruited prospectively, independently, and from a different study site after the confirmatory sample was completed. Sepsis was diagnosed according to the 2016 Sepsis definition [15] and by using all available information including imaging, antibiotic response and surgical findings; the final diagnosis of sepsis was made by experienced ICU clinicians without knowledge of the results of the progranulin test. Additionally, 182 ICU patients with sterile inflammatory reactions (SIRS) defined according to Sepsis-2 criteria [16] after open-heart surgery, 48 ICU patients with severe localized infections (e.g., large peripheral abscesses) at high risk for sepsis, 53 healthy volunteers, 31 patients with community-acquired pneumonia and 22 patient with COVID-19 associated pneumonia were recruited for the study to quantify the ability of progranulin to discriminate between these disease states. Community-acquired pneumonia was defined as an acute infection of the pulmonary parenchyma in a patient who has acquired the infection in the community and has not had recent hospitalization or association with other healthcare facilities such as nursing homes, dialysis centers, and outpatient clinics. A CURB-65 score ≥ 1 was required for the diagnosis of community-acquired pneumonia. Patients with malignancies, severe comorbid metabolic or cardiovascular disorders or transplantation as risk factors for community-acquired pneumonia were excluded (an overview of inclusion and exclusion criteria for the study is given in Table 1 and a flowchart of patient inclusion is shown in Fig. 1.

**Table 1:** Study inclusion and exclusion criteria according to groups

Criteria	Sepsis	SIRS	Pneumonia	Localized infection	Healthy controls
Inclusion	Sepsis or septic shock according to SEPSIS-3 criteria [15]	Systemic inflammatory response after major surgery and cardiopulmonary bypass (modified SEPSIS-2 criteria)	ICU or IMC patients with community-acquired pneumonia without recent hospitalization or association with other healthcare facilities such as nursing homes, dialysis centers, and outpatient clinics Clinical symptoms like fever, cough or dyspnea and a CURB-65 score ≥ 1 were required for diagnosis	ICU/IMC patients with large peripheral tissue infections (e.g., abscess formation after i.v. drug abuse)	Charlson Comorbidity Index = 0 [41]
Exclusion	No consent given by patients or next-of-kin				
	Age < 18				
	Pregnancy				
	Preexisting chronic infectious disorders (e.g., endocarditis, HIV or hepatitis)				
	Current tumor or malignant disorders				
	Limited patient’s life expectancy < 6 months (independent of, e.g., sepsis/SIRS, pneumonia or localized infection)				
	Immunosuppression^a^				

*ICU*  intensive care unit, *IMC* intermediate care

^a^Patients being on steroid therapy, autoimmune disease, transplantation or other immunocompromising conditions like HIV or leukemia

The performance characteristics of progranulin as a possible biomarker were compared to the standard reference biomarkers procalcitonin, C-reactive protein and interleukin-6 in all groups.

Consecutive patients with sepsis for the exploratory cohort were enlisted from participants of the Placebo-Controlled Trial of Sodium Selenite and Procalcitonin Guided Antimicrobial Therapy in Severe Sepsis (SISPCT) [17] studied in the Munich academic study center; patients for the confirmatory group were recruited from the Munich center (*n* = 44) and the Munich City Hospital (*n* = 83).

Patients for molecular network analysis were randomly selected from the confirmatory study group. Seven patients with sepsis and 6 healthy controls were included for initial high-throughput sequencing analysis (NGS), and 40 patients from the sepsis cohort and 23 volunteers were recruited for RT-qPCR confirmation.

### Sample collection and biochemical analysis

Progranulin and procalcitonin plasma concentrations were measured using commercially available ELISA tests (progranulin: MEDIAGNOST, Reutlingen, Germany; procalcitonin: Brahms Procalcitonin Assay, Thermo Fisher Diagnostics GmbH, Hennigsdorf, Germany). Interleukin-6 levels were quantified using the Multiplex Hybcell Technology (Cube Dx GmbH, 4300 St. Valentin, Austria).

Blood sampling and recording of clinical data were performed at admittance to the ICU and on day 1, 4, 7, 10, 14 and day 21 of ICU treatment or upon death, whichever came first. For differentiation between surgically induced SIRS and sepsis, blood samples drawn at admittance to the ICU after cardiac surgery were compared to samples obtained at admittance to the ICU in sepsis patients.

Blood samples were drawn from healthy subjects via venipuncture and from patients through intravascular arterial catheters. Samples to obtain serum were collected into 9-ml serum tubes (S-Monovette, Sarstedt, Germany), allowed to clot for 30 min and subsequently centrifuged at 3.400×*g* for 10 min at 4 °C. Whole blood samples designated for extraction of cellular RNA were collected in RNA tubes (PAXgene, QIAGEN, Hildesheim, Germany). Serum aliquots and RNA tubes were stored at − 80 °C.

### Molecular analyses

Patients for molecular network analysis were randomly selected from the confirmatory study group. Seven patients with septic shock and seven healthy controls were included in an initial high-throughput sequencing (NGS) analysis using blood cell-derived miRNA and protein-coding transcripts (mRNA). Differential expression data for miRNAs and mRNAs were sent to Ingenuity Pathway Analysis (IPA, QIAGEN, Redwood, CA, USA), paired and molecular response networks were constructed for progranulin. Significantly regulated miRNAs and mRNAs from a selected antimicrobial network were validated by RT-qPCR in a new cohort of septic patients sampled upon admission to the ICU (*n* = 40 for miRNAs; *n* = 39 for mRNAs) as well as in 23 additional healthy volunteers (Additional file 1: Table S7 for demographic and clinical data of all subgroups). Patients from the NGS group had comparable progranulin concentrations to RT-qPCR confirmation patients (64.1 ng/ml, 95% CI = 60.1–69.6 vs. 52.5 ng/ml, 95% CI = 40.6–76.0, *p* = 0.201).

### High-throughput sequencing

For extraction of blood cell RNA, PAXgene blood tubes were processed with the PAXgene blood miRNA Kit (QIAGEN, Hildesheim, Germany) according to the manufacturer’s protocol. Integrity of total blood cell-derived RNA was assessed with the RNA 6000 Nano assay on the Bioanalyzer 2100 (Agilent Technologies, Waldbronn, Germany). RNA was quantified by spectrophotometry using a ND-1000 NanoDrop (Thermo Fisher Scientific, Darmstadt, Germany).

Blood cell-derived small RNA and mRNA from seven patients diagnosed with septic shock and seven healthy volunteers were analyzed by high-throughput sequencing. For small RNA-Seq, we used 200 ng of total RNA as starting material and prepared libraries as described elsewhere [18]. Libraries for mRNA-Seq were constructed from 100 ng total RNA. Briefly, mRNA was captured by Poly(A) enrichment, followed by library preparation using the NEBNext Ultra RNA Library Preparation Kit (New England BioLabs, Ipswich, MA, USA). Adaptor-ligated libraries were enriched and indexed through 13 cycles of PCR amplification with the use of NEBNext Dual Index Primers Set 1 (New England BioLabs, Ipswich, MA, USA). After purification, the quality of resulting libraries was assessed by capillary electrophoresis using the Agilent High Sensitivity DNA Kit (Agilent Technologies, Waldbronn, Germany). Lastly, libraries were quantified via RT-qPCR using the KAPA SYBR FAST qPCR Master Mix (Kapa Biosystems, Wilmington, MA, USA). Both miRNAs and mRNAs were sequenced using 50 single-end cycles on a HiSeq 2500 (Illumina Inc., San Diego, CA, USA). Sequencing data for miRNAs was processed as described previously [18] FASTQ files of mRNA sequencing files were imported directly into the Array Studio software v10.0.1.81 (QIAGEN, Cary, NC, USA) package for further data analysis. All FASTQ files were aligned to the gene model Ensembl.v88 and to the reference library Human B38 using the proprietary OmicSoft Aligner OSA [19]. Differential gene expression between patients and volunteers was assessed using DESeq2 [20]. For miRNAs, a log_2_-fold change ≥|1|, an adjusted *p*-value (*p*_adj_) of ≤ 0.05 and a mean expression of ≥ 50 were set as thresholds to identify significantly regulated transcripts. Differential expression data for protein-coding transcripts was sent directly to Ingenuity Pathway Analysis (IPA) for biological analysis using the cutoffs *p*_adj_  ≤ 0.05, log_2_-fold change ≥|1| and mean expression of ≥ 10 for subsequent pairing with differentially expressed miRNAs.

### RT-qPCR validation

A selection of differentially regulated miRNAs and mRNAs from NGS was further investigated by RT-qPCR in a new cohort of septic patients sampled upon admission to the ICU (*n* = 40 for miRNAs; *n* = 39 for mRNAs) as well as healthy volunteers (*n* = 23). Prior to RT-qPCR, we used geNorm [21] and NormFinder [22] to predict the most stably expressed miRNAs and mRNAs based on the NGS data set and selected potential reference candidates. Reverse transcription of miRNAs was carried out using 10 ng of total RNA and the miRCURY LNA RT Kit (QIAGEN, Hildesheim, Germany). Real-time PCR was performed using 3 µl of diluted cDNA and QIAGEN’s miRCURY LNA SYBR Green PCR Kit. For analysis of mRNAs, 300 ng of total RNA were reverse transcribed using the QuantiTect RT Kit (Qiagen, Hildesheim, Germany). We then used the Sso Advanced Universal SYBR Supermix and PrimePCR Assays (Bio-Rad, Munich, Germany) to quantify mRNA expression in 10 ng cDNA. All reactions were carried out in a 10 µl total reaction volume on a Rotor-Gene Q thermal cycler (QIAGEN, Hildesheim, Germany). Expression of miRNAs was normalized with the geometric mean of miR-625-3p, miR-501-3p and miR-30d-5p, while mRNAs were normalized with the geometric mean of Ribophorin I, Transmembrane BAX inhibitor motif containing 6 (TMBIM6) and Ubiquitin C. Relative quantification was carried out using the ΔΔCq method [23].

### Pathway analysis

Ingenuity Pathway Analysis (IPA, QIAGEN, Redwood, CA, USA) was used for the identification of miRNA and mRNA regulation and networks resulting from our high-throughput miRNA/mRNA expression data.

Significantly regulated miRNAs and mRNAs meeting the predefined cutoffs were entered into IPA microRNA Target Filter, and expression values were paired. Because of the important role of progranulin in the early response to microbial challenges [8, 9], IPA disease filtering was set to “antimicrobial response” in addition to “infectious disease”, and pathways related to autoimmune diseases were excluded. Only experimentally confirmed relationships were considered for the analysis of miRNA/mRNA interaction and the construction of transcriptomic networks.

### Statistical analysis

Sample size estimation for the comparison between sepsis patients and a non-infected group (SIRS) was based on an earlier study which evaluated progranulin levels in a total of 121 patients with suspected neonatal sepsis [24]. With this sample size, serum PGRN levels of the infected group were significantly higher than in the uninfected group and ROC areas under curve for progranulin, procalcitonin and interleukin-6 and were significant at a *p*-value < 0.001. Although our study was performed in adults, we selected sample sizes in the range of 120 patients for the exploratory and the confirmatory study samples.

Demographic characteristics and clinical data between all subgroups were compared using the Mann–Whitney *U* test or analysis of variance of ranks (ANOVA) followed by post hoc testing when more than two groups were present. The Chi-square or Fisher’s exact test was used for comparison of categorical variables. Data in the text and in tables are given as median and quartiles or range when more appropriate.

For evaluation of the predictive performance, we calculated area under the curve (AUC) values and constructed receiver-operating-characteristic (ROC) curves. This analysis was performed in the initial exploratory sample and in the following confirmatory study and the results compared. Exploratory cut-off values were obtained by calculating the Youden Index [25] which was optimized iteratively to reach a minimal sensitivity of > 80% and a maximal specificity. Positive (LR +), negative (LR−) likelihood ratios and positive (PV +) and negative (PV−) predictive values were given for cut-off values. AUC values for progranulin and the reference biomarkers procalcitonin, C-reactive protein and interleukin-6 were compared using a pairwise AUC analysis by the method of DeLong et al. [26]. This analysis was performed in both subgroups separately and the results were compared.

Indeterminate or missing results from biomarkers (progranulin, procalcitonin, C-reactive protein and interleukin-6) were individually discarded during the statistical analysis.

All statistical tests were two-tailed and a *p*-value < 0.05 was considered statistically significant. Data analysis was performed using Python Version 3.7 (Python Software Foundation, Beaverton, OR, USA) and SigmaPlot Version 12.5 (Systat Software, Inc., San Jose, CA, USA). The following libraries were used in Python in order to calculate mean, median, quartiles, Mann–Whitney *U*, Chi-square test, ROC curves, and to plot graphics: Numpy, Pandas, Scipy, Matplotlib, Seaborn, Sklearn.

## Results

### Study populations of the exploratory and the confirmatory sepsis sample

The incidence of septic shock among the patients with sepsis was 65.8% in the exploratory group and 73.3% in the confirmatory group (*p* = 0.265). Mortality was 18.4% and 22.8% (*p* = 0.494), respectively. No difference was seen in progranulin plasma concentrations at study inclusion between both cohorts (60.2 ng/ml, Q25–Q75: 44.5–89.4 vs. 56.0 ng/ml, Q25–Q75: 42.0–78.0, *p* = 0.176) and the other biomarkers used for comparison (see Additional file 1: Table S1).

### Progranulin and reference biomarker plasma concentrations

**Fig. 2:**
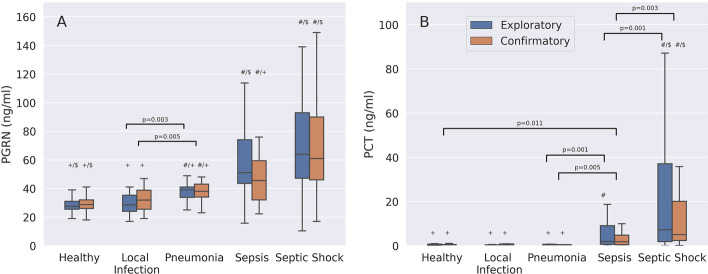
Comparison of progranulin (PGRN, **A**) and procalcitonin (PCT, **B**) plasma concentrations between healthy controls and patients with either a severe localized infection (e.g., a large peripheral abscess at high risk for sepsis), non-COVID-19 community-acquired pneumonia (CAP), sepsis or septic shock. Data are presented separately for the exploratory (blue boxplots) and the confirmatory study (orange boxplots). All symbols indicate *p* < 0.001. Significant *p*-values ≥ 0.001 are given as numbers in addition to symbols. # Indicates a significant difference between patients with sepsis, septic shock or CAP when compared to healthy controls. $ Indicates a significant difference between patients with sepsis, septic shock or healthy controls when compared to patients with CAP. + Indicates a significant difference between patients with sepsis, CAP or healthy controls when compared to septic shock patients

In both sepsis groups combined (*n* = 241), progranulin concentrations were 57.5 ng/ml (Q25–Q75: 42.8–84.9) and significantly higher than in healthy individuals (*n* = 53) (28.7 ng/ml (Q25–Q75: 25.5–31.7, *p* < 0.001) (Additional file 1: Table S5). Patients with non-COVID-19 CAP showed significantly higher progranulin plasma levels than healthy controls in both the exploratory and the confirmatory group (Fig. 2A). The progranulin plasma concentrations were also significantly higher in patients with non-COVID-19 CAP than in individuals with a localized, peripheral infection in both study cohorts. Individuals with COVID-19 pneumonia showed significantly higher progranulin plasma concentrations than patients with non-COVID-19 CAP (67.6 (Q25–Q75: 56.6–96.0) vs. 38.0 (Q25–Q75: 33.5–41.0) ng/ml, *p* < 0.001). Demographic and clinical data between COVID-19 and non-COVID-19 CAP patients are compared in Additional file 1: Table S10. Patients with sepsis only (not septic shock) had higher progranulin plasma concentrations than patients with non-COVID-19 CAP, but the difference was only significant in the exploratory group (Fig. 2A). Progranulin plasma concentrations correlated with SOFA scores (*r* = 0.353, *p* = 1.33E−7, *n* = 241).

In contrast to progranulin, procalcitonin levels were not significantly different between healthy controls and patients with either a localized infection or non-COVID-19 CAP (both groups, *p* ≥ 0.195), but were marginally but significantly higher when COVID-19 patients were compared to those with non-COVID-19 CAP (Additional file 1: Table S10). Procalcitonin concentrations were significantly higher in septic shock patients compared to the other study groups in both the exploratory and confirmatory study (Fig. 2B). Unlike progranulin, procalcitonin did not show significant differences in plasma levels between patients with localized infections or non-COVID-19 CAP (Fig. 2B). Progranulin and procalcitonin plasma concentrations correlated significantly (Spearman’s rho = 0.472, *p* = 2.015E−11, *n* = 181).

### Progranulin concentrations differ according to the primary septic focus

**Fig. 3:**
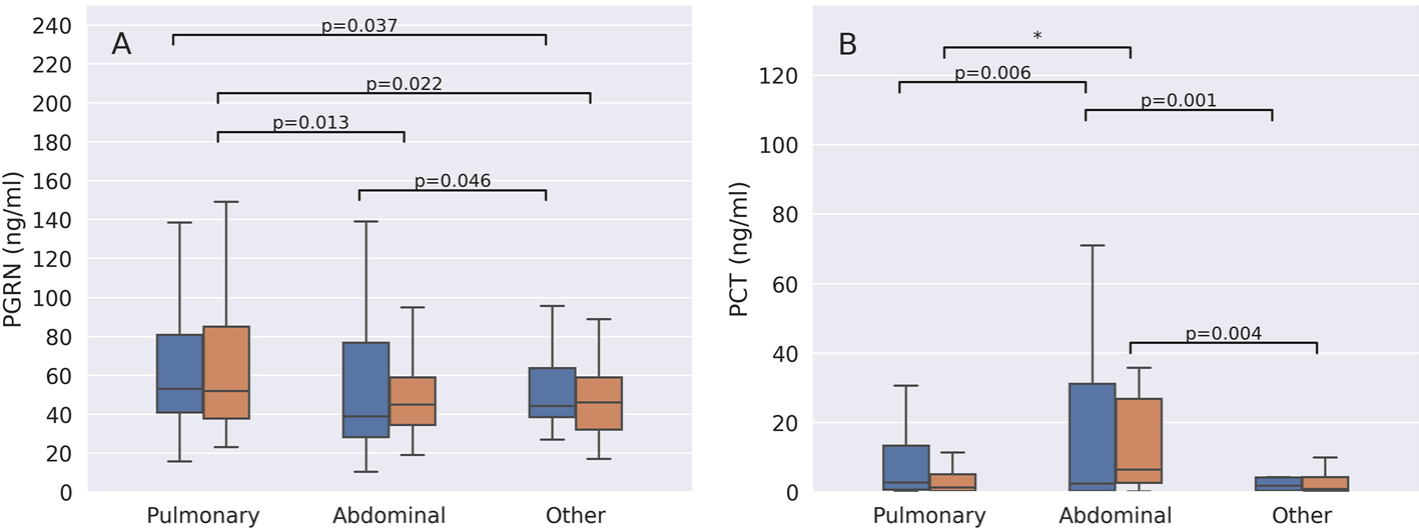
Comparison of progranulin (PGRN) (**A**) and procalcitonin (PCT) (**B**) plasma concentrations in sepsis of pulmonary, abdominal or other origin. Data are presented separately for the exploratory (blue boxplots) and the confirmatory study (orange boxplots). Significant differences between groups are marked with bars and *p*-values. * Indicates a *p* value < 0.001

When patients were grouped according to the presumed origin of sepsis (pulmonary, abdominal or other locations), those with a pulmonary origin of infection had significantly higher progranulin concentrations than patients with abdominal infection (only in the confirmatory study, *p* = 0.013) or other causes of sepsis (*p* = 0.037 exploratory and *p* = 0.022 in the confirmatory group) (Fig. 3A). In contrast to progranulin, procalcitonin values were significantly lower in pulmonary or other causes of sepsis than in abdominal infections (Fig. 3B).

### Progranulin concentration at baseline is associated with ICU mortality

Patients who died during ICU therapy had significantly higher progranulin plasma concentrations at ICU admission than survivors (73.7 ng/ml, Q25–Q75: 50.4–104.5 vs. 55.7 ng/ml, Q25–Q75: 41.0–76.2, *p* = 0.004), whereas plasma levels of procalcitonin, C-reactive protein and interleukin-6 were not significantly different between both groups (Additional file 1: Table S4).

### Progranulin concentrations remain elevated during ICU therapy

**Fig. 4:**
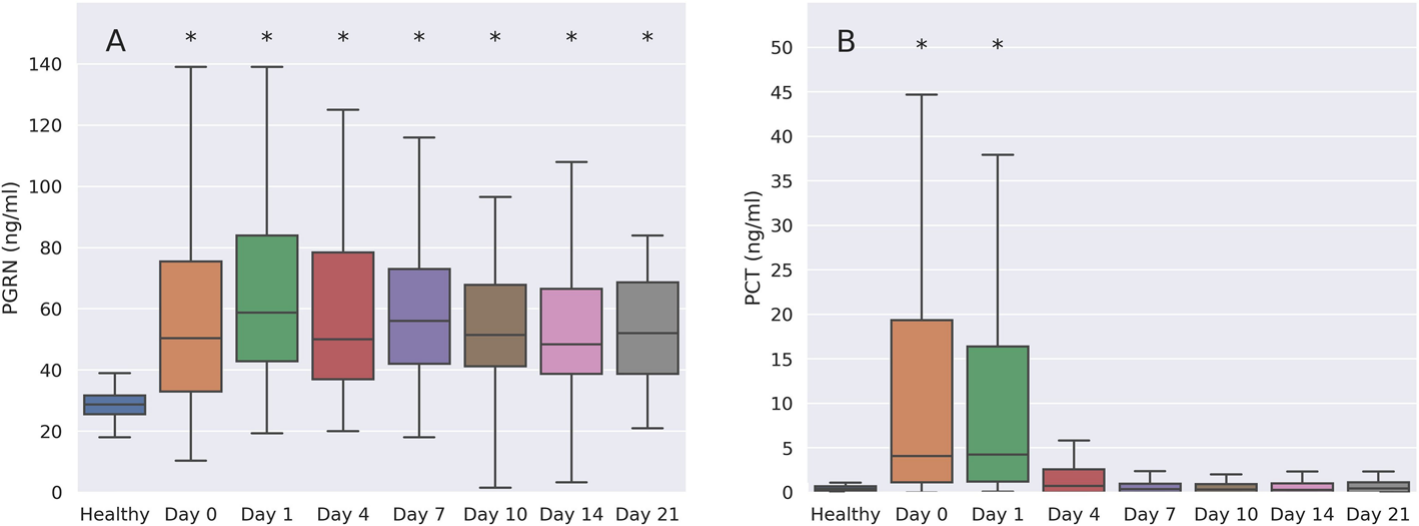
Kinetics of progranulin (PGRN, **A**) in comparison to procalcitonin (PCT, **B**) over the course of ICU therapy from day 0 (ICU admittance and study inclusion) until day 21 in surviving patients (*n* = 191). Both the exploratory and the confirmatory group were pooled for this illustration. * Indicates a *p* < 0.001 (ANOVA on ranks with Dunn’s post hoc test)

Progranulin and procalcitonin plasma concentrations showed different kinetics during ICU therapy. In patients surviving until day 21 of ICU therapy (*n* = 191, both study groups), progranulin remained significantly elevated, whereas procalcitonin levels declined significantly over time (Fig. 4).

### Progranulin plasma levels are raised by infection, but not inflammation per se

**Fig. 5:**
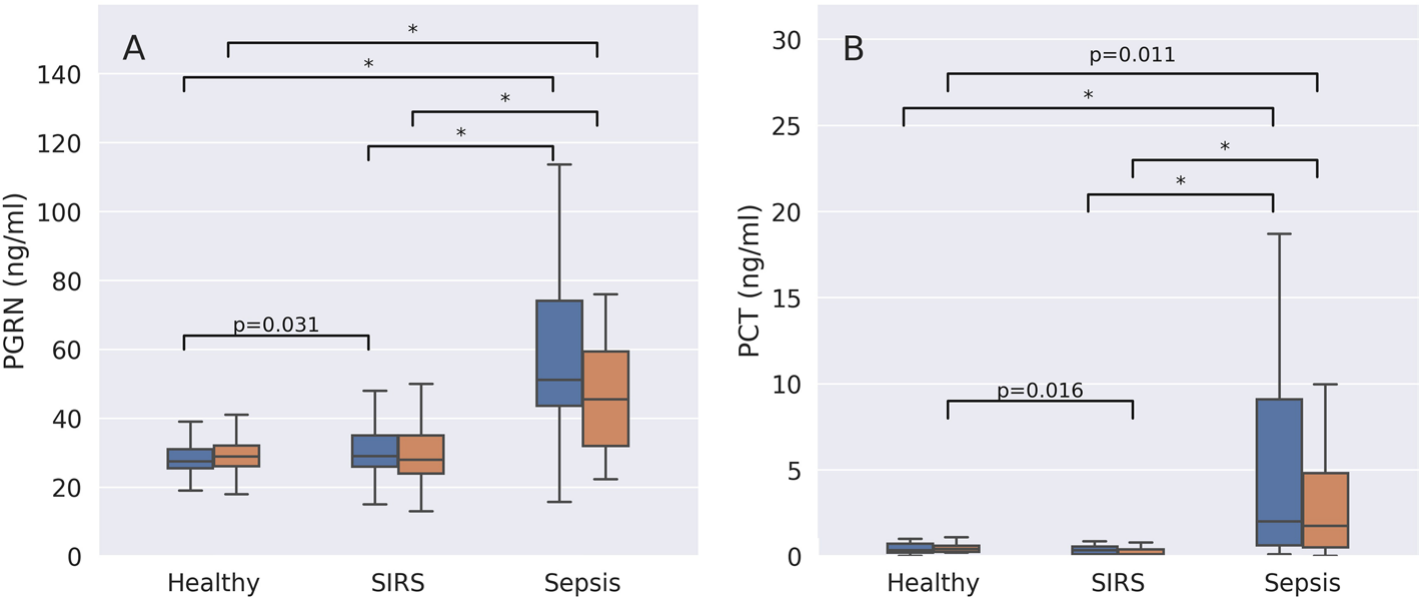
Comparison of progranulin (PGRN, **A**) and procalcitonin (PCT, **B**) plasma concentrations in healthy volunteers, patients with SIRS and patients with either sepsis or septic shock ("Sepsis") at study inclusion. Data are presented separately for the exploratory (blue boxplots) and the confirmatory study (orange boxplots). *Indicates a *p* < 0.001, significant *p* values ≥ 0.001 are given as numbers

Plasma concentrations of progranulin and all reference biomarkers were significantly higher in sepsis than in SIRS. Of note, progranulin concentrations were only significantly different between healthy individuals and patients with SIRS in the exploratory cohort, whereas procalcitonin (only in the confirmatory cohort), C-reactive protein and interleukin-6 levels were significantly higher in SIRS than in healthy controls (Fig. 5. see Additional file 1: Table S3 for CRP and IL-6 plasma concentrations), which indicates that progranulin plasma levels may be less responsive to inflammation in the absence of microbial infection than C-reactive protein and interleukin-6.

### Diagnostic performance of progranulin in comparison to reference biomarkers

#### Differentiation between sepsis and SIRS

**Fig. 6:**
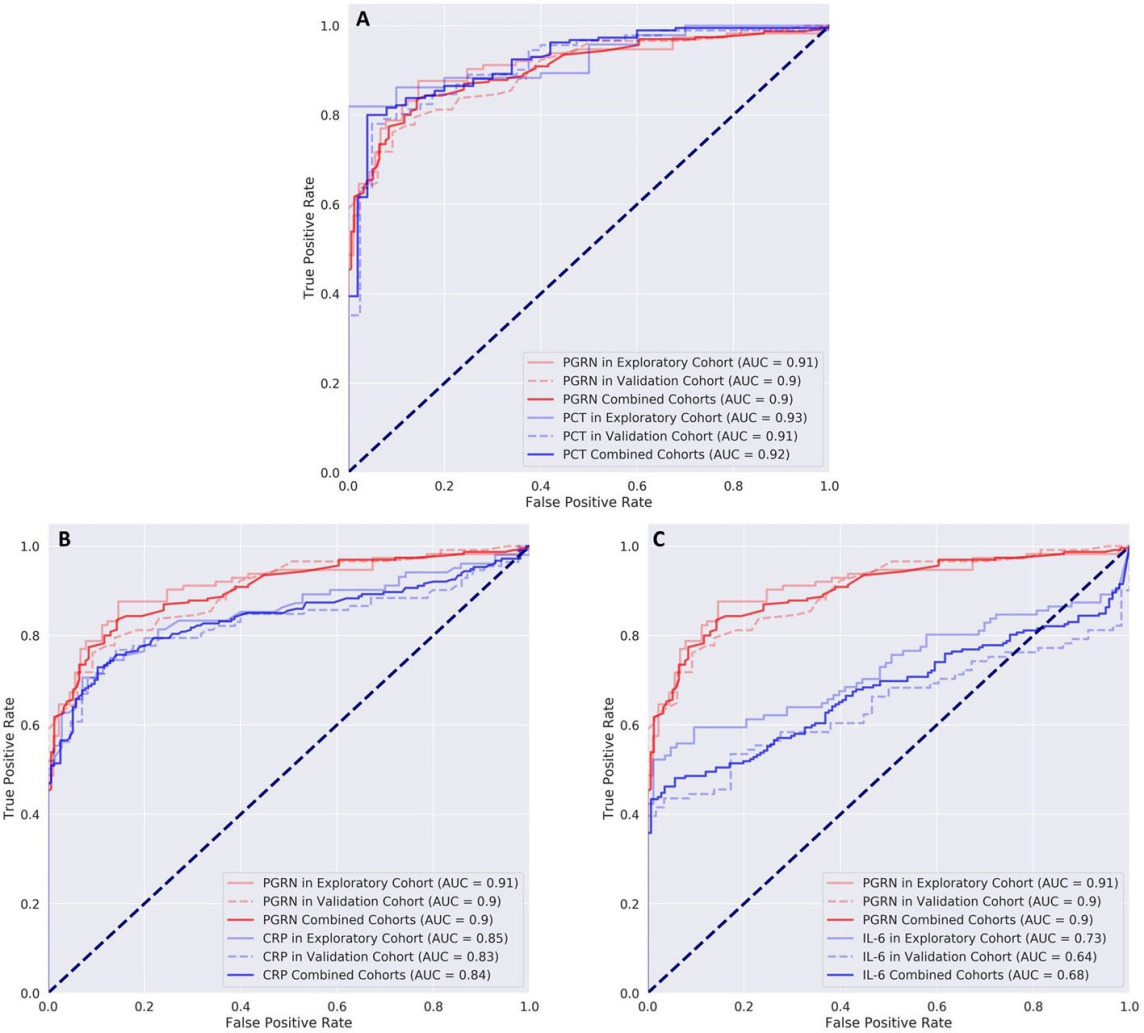
ROC curves for the differentiation between patients with SIRS and sepsis for procalcitonin (PCT, **A**), C-reactive protein (CRP, **B**) and interleukin-6 (IL-6, **C**) in comparison to progranulin. Patients with sepsis and septic shock were combined for this analysis. The curves for progranulin are printed in red and the respective lines of the compared reference marker are outlined in blue. Lines printed in lighter colors represent measurements in the exploratory cohort and dashed lines the corresponding measurements in the confirmatory (validation) sample. Dark lines show the summary values from both samples. See text for AUC values and statistical comparison and Additional file 1: Tables S8 and S9 for further details

The diagnostic ability of progranulin to distinguish between sepsis and SIRS was comparable to that of procalcitonin in both the exploratory and the confirmatory sample (Fig. 6). When both samples were combined (*n* = 423 patients in total), AUC for identifying patients with SIRS but not sepsis was 0.90 (95% CI = 0.87–0.93) for progranulin and 0.92 (95% CI = 0.88–0.96, *p* = 0.323) for procalcitonin. The combined AUC value for procalcitonin and progranulin was 0.94 and thus only marginally better than those of the individual biomarkers. For the individual cut-off values please see Additional file 1: Table S9. Additional file 1: Tables S8 and S9 compare the corresponding AUC values for C-reactive protein and interleukin-6 as well as cut-off values of all tested biomarkers according to subgroups. When testing the validation cohort using the cut-off values for progranulin and procalcitonin derived from the exploratory cohort, no significant differences between progranulin and procalcitonin were found (progranulin odds ratio: 24.11 (95% CI 10.46–55.56), procalcitonin odds ratio 63.33 (95% CI 14.09–284.75), Breslow–Day test for significant difference *p* = 0.259).

More statistical details including likelihood ratios and probabilities are also given in the Additional file 1.

#### Differentiation between localized infections and sepsis

The comparison of patients with severe localized infections and sepsis showed no significant difference in diagnostic performance between progranulin and procalcitonin in both the exploratory and the confirmatory group (Additional file 1: Figure S4). AUC values for progranulin in the combined sample (241 patients with sepsis and 48 patients with localized infection) were 0.90 (95% CI = 0.86–0.94) and 0.89 (95% CI = 0.84–0.94) for procalcitonin (*p* = 0.790) for the identification of patients with localized infections in the absence of sepsis. Combining both biomarkers did not improve the overall predictive performance for sepsis (AUC = 0.85). Progranulin and procalcitonin were superior to interleukin-6 but not better than C-reactive protein (Additional file 1: Figures S4B, C and Table S8). Testing the odds ratio of the validation cohort with the cut-off values of the exploratory cohort did not yield significant differences between progranulin and procalcitonin (*p* = 0.894). More details of this analysis are also given in the Additional file 1.

#### Diagnostic performance of progranulin in pneumonia

**Fig. 7:**
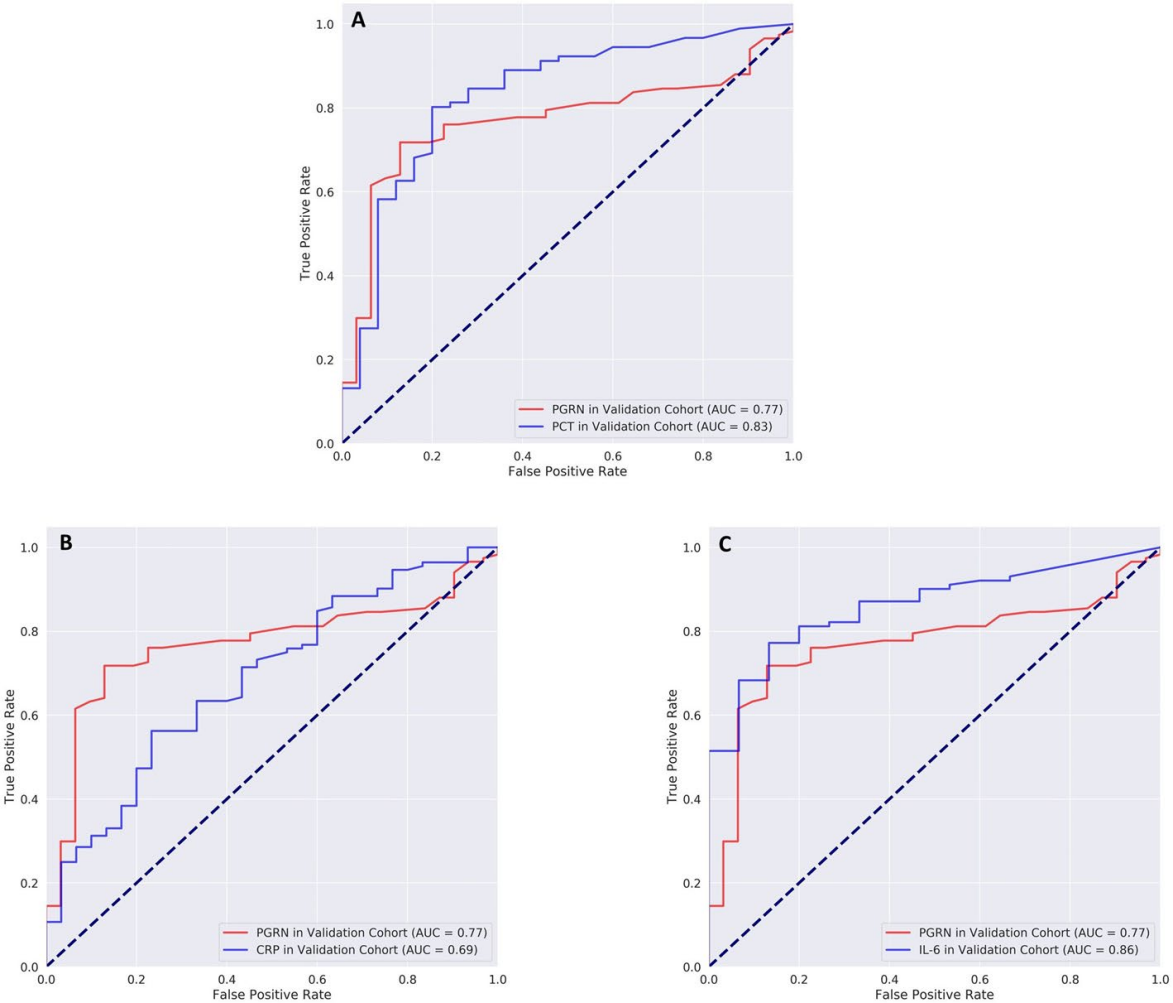
ROC curves for the differentiation between patients with community-acquired pneumonia (CAP) and patients with sepsis in the validation cohort. Red lines indicate ROC curves for progranulin in all graphs and blue lines the corresponding values for procalcitonin (PCT, **A**), C-reactive protein (CRP, **B**) and interleukin-6 (IL-6, **C**). Cut-off values and data for sensitivity/specificity for the reference biomarkers in comparison to progranulin are given in Additional file 1: Table S9

Progranulin differentiated well between healthy volunteers (*n* = 53) and patients with non-COVID-19 CAP (*n* = 31) (AUC = 0.87, 95% CI = 0.77–0.94, sensitivity = 83.9%, specificity = 82.4%^1^ (Tables S8, S9). For the differentiation between sepsis in the confirmatory (*n* = 127) and non-COVID-19 CAP (*n* = 31), AUC values were 0.77 (95% CI = 0.68–0.85) for progranulin and 0.83 (95% CI = 0.73–0.92, *p* = 0.36) for procalcitonin. Combining both biomarkers did not improve diagnostic performance (AUC = 0.87). Cut-off values for progranulin were 38.0 ng/ml at a sensitivity of 81.2% but with low specificity (45.2%). The corresponding values for procalcitonin were 0.79 ng/ml (sensitivity = 80.2%, specificity = 80.0%). AUC values for procalcitonin and interleukin-6 were higher than for progranulin and lower for C-reactive protein but these differences were not statistically significant (*p* ≥ 0.06) (see Fig. 7 and Additional file 1: Tables S8 and S9 for more details).

**Fig. 8:**
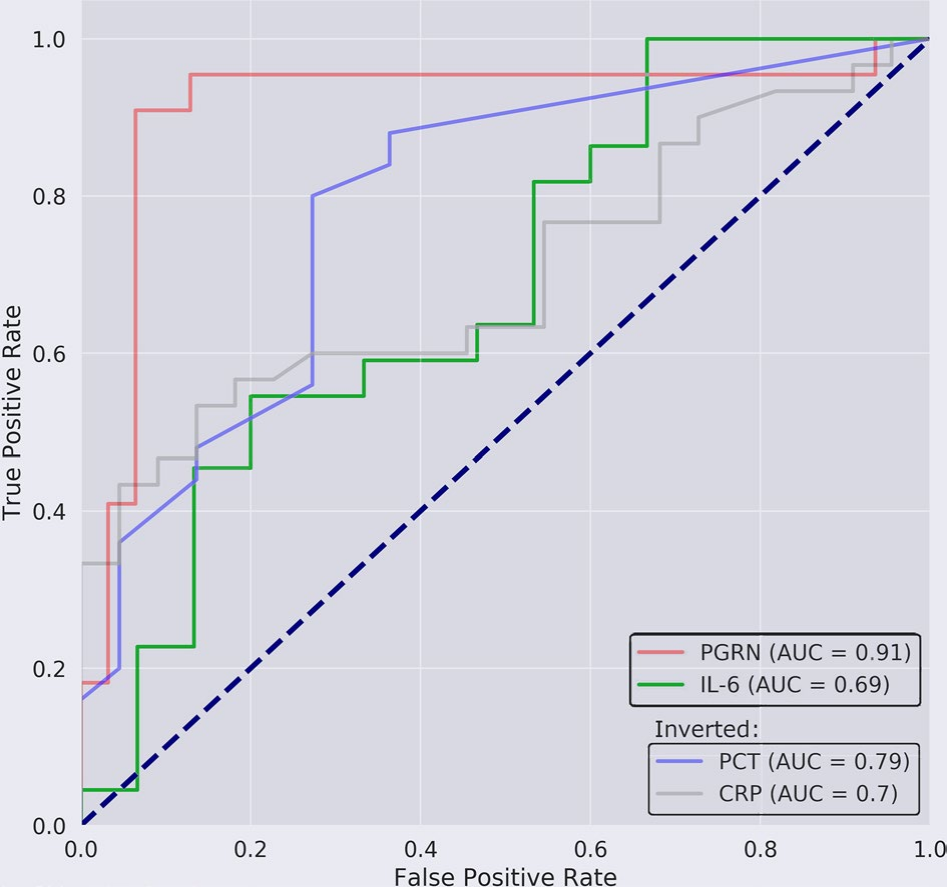
ROC analysis for the differentiation between patients with viral COVID-19 or non-COVID-19 pneumonia (CAP) for progranulin vs. procalcitonin (PCT), interleukin-6 (IL-6) or C-reactive protein (CRP). Procalcitonin and C-reactive protein act as a negative predictor for the detection of a SARS-COV-2 pneumonia and have therefore been inverted for this ROC analysis

In contrast, in patients with COVID-19 pneumonia, procalcitonin and C-reactive protein were negative predictors for COVID-19 pneumonia, whereas progranulin and interleukin-6 were positive predictors of COVID-19. All biomarker concentrations differed significantly between viral and non-COVID-19 pneumonia (Additional file 1: Table S10), but progranulin was significantly better in differentiating between COVID-19 and non-COVID-19 pneumonia (AUC = 0.91, 95% CI = 0.8–1.0 for progranulin vs. AUC = 0.79 for procalcitonin with 95% CI = 0.66–0.92 *p* =  < 0.001, see Table S8 and Fig. 8) and between COVID-19 and healthy individuals (AUC = 0.97, 95% CI = 0.91–1.03). The threshold value of progranulin for the differentiation between COVID-19 and non-COVID-19 pneumonia yielded a sensitivity of 0.91 (95% CI = 0.71–0.99) at a specificity of 0.94 (95% CI = 0.79–0.99) with a positive likelihood ratio (LR^+^) of 14.09, a negative LR^−^ of 0.10, a positive predictive value (PV^+^) of 0.91 and negative PV^−^ of 0.93. The corresponding values for COVID-19 vs. the healthy state were sensitivity = 0.91 (95% CI = 0.71–0.99), specificity = 1.00 (95% CI = 0.93–1.00) with LR^−^ = 0.09, PV^+^ = 1.00 and PV^−^ = 0.96. The dot histograms in Additional file 1: Figure S5 illustrate the superior diagnostic performance of progranulin in comparison to the other biomarkers for this differentiation in detail.

Ten patients from the COVID-19 group needed later transfer to the ICU as they developed ARDS requiring intubation and mechanical ventilation. When comparing biomarkers between COVID-19 patients who developed ARDS to those not requiring ICU treatment, progranulin and interleukin-6 were the only biomarkers to differ significantly (*p* < 0.001) on the day of admission to the hospital (see Additional file 1: Table S11).

#### Mortality prediction by progranulin

Mortality prediction for all tested biomarkers was poor in both the exploratory and the confirmatory group (see Additional file 1: Tables S8 and S9). When the exploratory and the confirmatory sample were combined, AUC values were 0.63 (95% CI = 0.54–0.72) for progranulin and 0.56 (95% CI = 0.46–0.66, *p* = 0.143) for procalcitonin. The combination of both progranulin and procalcitonin did not result in better prognostic performance for ICU mortality (AUC = 0.36).

Testing the odds ratio of the validation cohort with the cut-off of the exploratory cohort did not yield a significant difference in the odd-ratios between progranulin and procalcitonin (*p* = 0.885).

### Molecular network of progranulin regulation

NGS in patients with septic shock in comparison to healthy volunteers revealed 82 significantly regulated miRNAs (40 upregulated) and 2918 significantly changed mRNAs (1385 upregulated). Significantly upregulated mRNAs from blood cells included progranulin (GRN) (mRNA *GRN* log_2_FC = 2.23,* p*_adj_ = 3.46E−8) and, interestingly, the progranulin functional regulator sortilin [27] (*SORT1*) (mRNA *SORT1* log_2_FC = 5.56, *p*_adj_  = 1.38E−8), both of which could be confirmed by RT-qPCR. NGS did not detect any transcripts of calcitonin gene (*CALC1*) mRNA as a potential source of circulating procalcitonin (PCT) in sepsis [28].

**Fig. 9:**
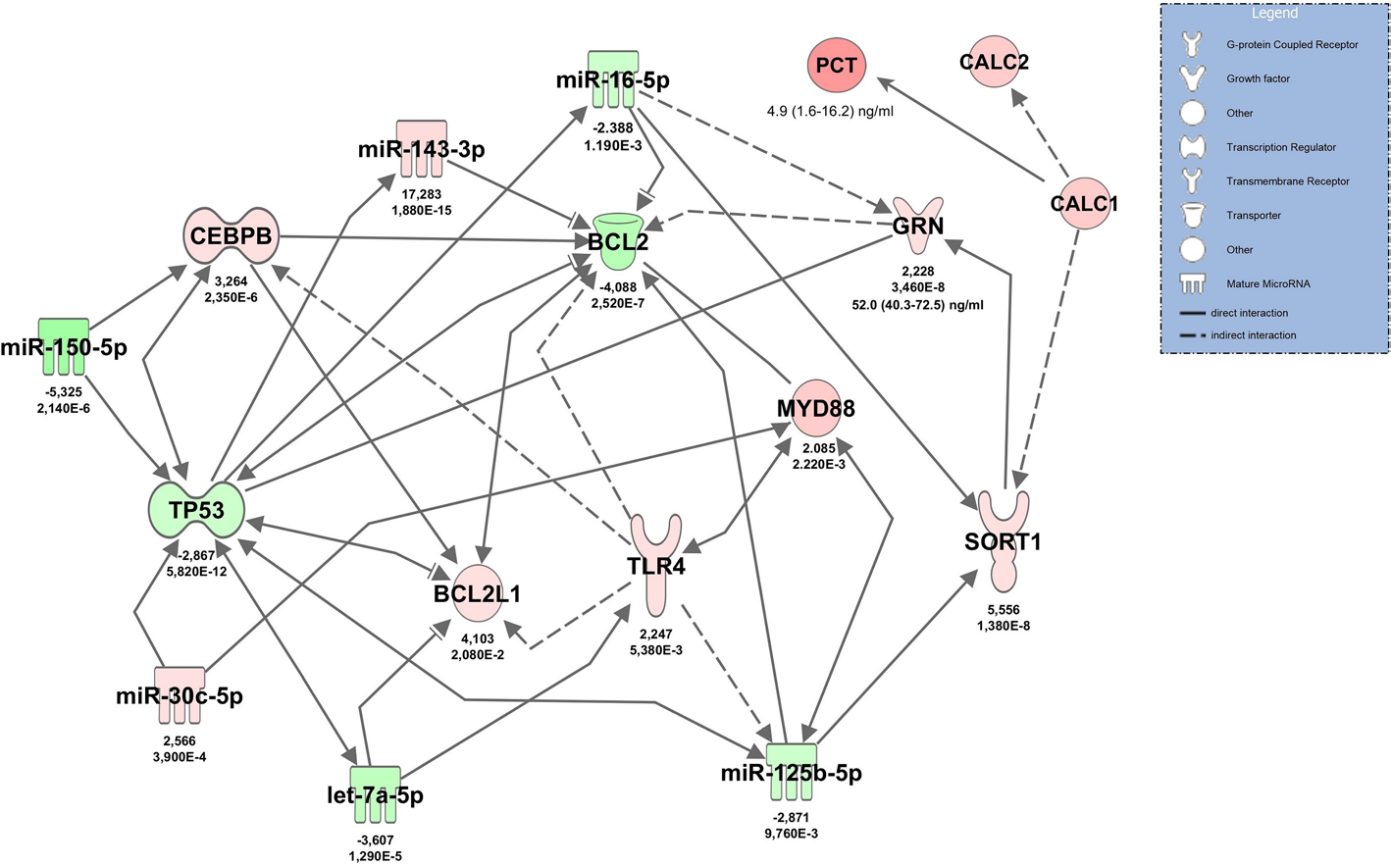
Illustration of the role of progranulin (GRN gene) in the molecular network activated during the early antimicrobial response in patients with septic shock at admittance to the ICU. The network was constructed using data from high-throughput sequencing followed by RT-qPCR confirmation. Red color indicates upregulation in comparison to healthy volunteers, green indicates downregulation. Color shading corresponds to log2FC values, which are reported below the molecules along with FDR-adjusted *p*-values. The effect of sepsis-associated upregulation of calcitonin precursors (CALC1) and its gene product procalcitonin on sortilin (SORT1), the principal binding partner of progranulin, is illustrated on the right side of the graph

Filtering and pairing of NGS miRNA/mRNA data for early antimicrobial response revealed a network including tumor suppressor protein p53 (*TP53*) and *TLR4* as well as upregulated *GRN* and *SORT1*, regulated by miR-16, miR-150 and others (Fig. 9). Apart from (mRNA) *BCL2L1*, all miRNAs and mRNAs in this network could be confirmed by RT-qPCR. Within the RT-qPCR confirmation group, normalized Cq-values for (cDNA) *GRN* (rho = 0.539, *p* < 0.001, *n* = 39) and (cDNA) *SORT1* (rho = 0.404, *p* = 0.006, *n* = 39) correlated with progranulin (GRN) plasma levels. (mRNA) *SORT1* expression levels also correlated with other key molecules in the network (e.g., TLR4: rho = 0.390, *p* = 0.007; BCL2: rho = − 0.730, *p* < 0.001; TP53: rho = − 0.749, *p* < 0.001), whereas GRN did not (data not shown).

Because the combination of GRN and procalcitonin did not improve the overall diagnostic performance in comparison to that of the single agents, we integrated PCT and its precursor *CALC1* into the antimicrobial response network in order to demonstrate possible interactions of both biomarkers. The model identified upregulated SORT1 as a target of GRN, resulting in increased binding of GRN protein and a possible reduction in free GRN levels with increased PCT concentrations (Fig. 9).

**Fig. 10:**
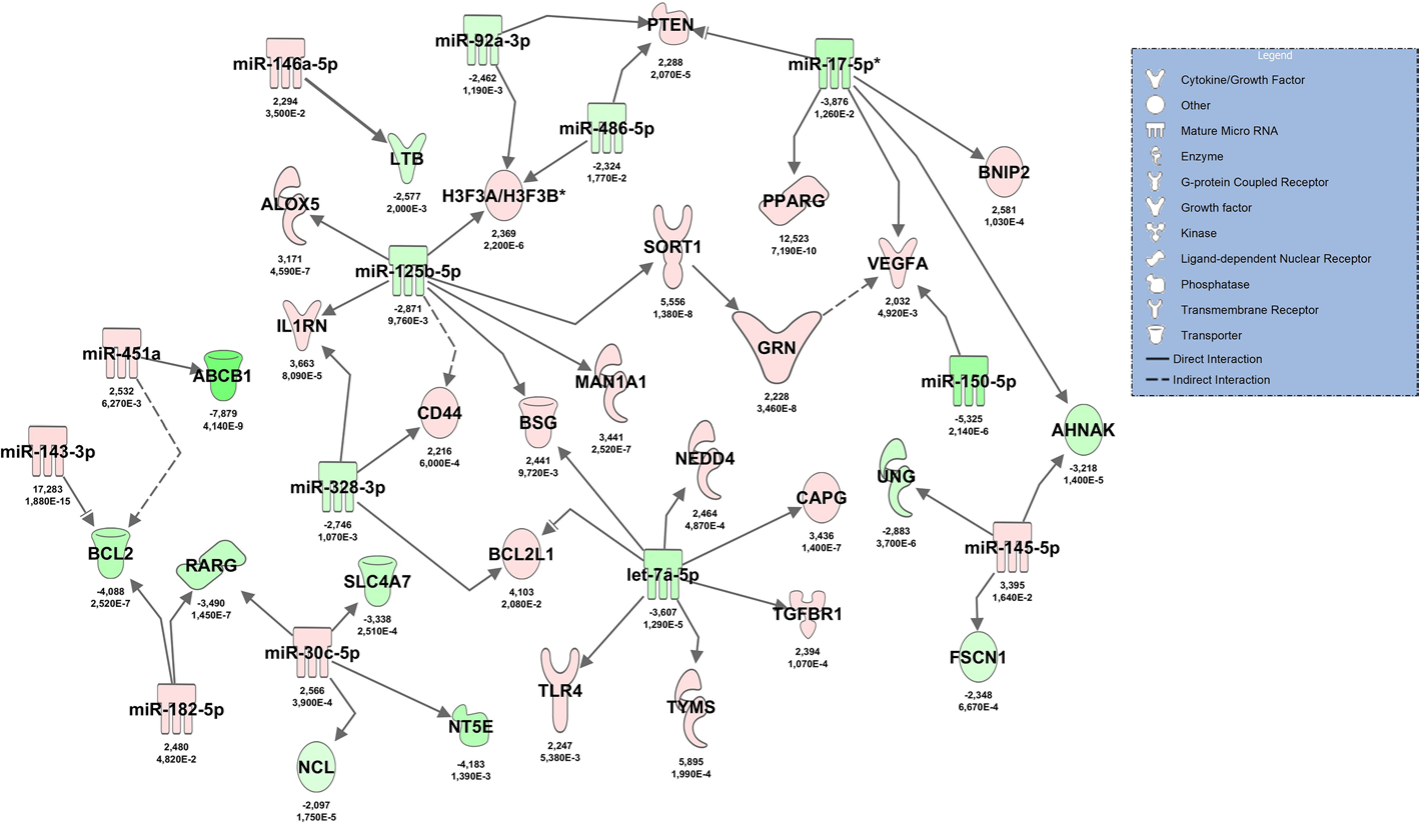
Molecular network constructed from high-throughput sequencing data showing progranulin activation during the early inflammatory response of immune cells during sepsis. Red color indicates upregulation in comparison to healthy volunteers, green indicates downregulation. Color shading corresponds to log_2_FC values. These values are indicated below the molecules along with FDR-adjusted *p*-values. Molecules are shown as symbols in the network, and the molecule type is given in the legend

As GRN is known to be activated in acute inflammation, we performed additional pathway analyses based on NGS data to delineate an additional network involved in the inflammatory response of immune cells. This network demonstrated an upregulating effect of miR-125b-5p on SORT1 and GRN, and identified vascular endothelial growth factor A (VEGFA) as a possible target of GRN in sepsis. These networks are shown in Fig. 10.

## Discussion

We investigated the functional role of the pleiotropic growth factor progranulin in two large cohorts of patients with sepsis and compared progranulin plasma levels between sepsis, SIRS, severe localized infections, community-acquired bacterial pneumonia and COVID-19. These comparisons demonstrated statistically significant differences in plasma concentrations between these disorders, significant relationships between disease severity and progranulin concentrations and identified an important role of progranulin signaling in the early antimicrobial response in sepsis. These findings provided evidence for the logic validity of using progranulin as a biomarker for sepsis and pneumonia and for the differentiation between these disorders.

We then tested this assumption in two large cohorts of patients, with the conditions SIRS, and severe localized infections as distractors. The diagnostic performance of progranulin to differentiate between sepsis and the distractors was comparable to that of procalcitonin, and generally better than the proinflammatory markers C-reactive protein and interleukin-6. Progranulin plasma levels also showed good diagnostic capabilities for the differentiation between patients with CAP and healthy individuals but performed worse for the separation between CAP and sepsis, although the differences in AUC values between progranulin and procalcitonin were not statistically significant.

We have to point out, however, that our study was not designed as a classic biomarkers study in the true sense of the definition of a validated biomarker [29] but intended as a combination of functional molecular findings with progranulin-related outcome data to demonstrate the soundness of studying progranulin as a future biomarker. In this context it is important to stress that in the clinical setting a physician usually does not solely rely on a singular biomarker, but rather on a combination of findings. Biomarker play an important role in diagnostics, but only in combination with other factors. Although we included a high number of sepsis patients and confirmed our findings in two different and independent study populations and used extensive and accepted statistical methods, our study still has limitations in case numbers, particularly in the sample sizes of the distractor “localized infection” and pneumonia including the COVID-19 group. This issue needs to be addressed in further validation studies for progranulin as an additional and novel biomarker.

A further limitation of our study results from the fact that in clinical reality, sepsis prevalence in patients with the distractor conditions SIRS, localized infection and CAP is generally lower than in our investigations. This results in higher pretest probabilities which could artificially inflate AUC values and sensitivity/specificity of the index test. Furthermore, our control patients with SIRS consisted exclusively of patients after cardiac surgery with potentially specific effects of heart disease and cardiopulmonary bypass on the index test. These facts should be regarded as a further shortcoming of our study.

When progranulin plasma concentrations were analyzed according to the presumed origin of sepsis, patients with CAP had significantly higher progranulin levels than study participants with an abdominal or other septic focus. The specific role of progranulin in pulmonary immunity has been demonstrated in a number of experimental studies [30–32], and elevated levels of progranulin in humans with CAP have also been shown in a previous small investigation [30]. Furthermore, and quite surprisingly, progranulin showed high sensitivity and specificity to discriminate between COVID-19 and non-COVID-19 CAP, and healthy individuals. This finding is mainly of pathophysiologic interest, as more sensitive molecular tests for COVID-19 are available (PCR) but points to an important regulatory role of progranulin in viral and non-viral pneumonia which has also been confirmed in several recent publications [14, 30, 32].

Progranulin and procalcitonin plasma concentrations showed different kinetics during ICU therapy. Whereas procalcitonin levels declined significantly within 48 h after admittance indicating a therapeutic response, progranulin concentrations remained elevated in surviving patients until day 21. This may indicate that procalcitonin, with a half-life of approximately 24 h, is more of an indicator of tissue hypoperfusion and inflammation during shock states [33] whereas progranulin is more sensitive in signaling ongoing immune activation in survivors of sepsis in the ICU. This line of reasoning is corroborated by our observation that progranulin (GRN gene transcript) expression was upregulated in blood cells in patients at admittance to the ICU, whereas no transcripts of CALC1, the gene coding for calcitonin could be detected. Bacterial infection is known to cause an upregulation of CALC1 gene expression and a consecutive release of procalcitonin from nearly all septic tissues and multiple cells throughout the organism [28], but, as shown by our sequencing data, probably not from circulating blood cells.

Progranulin is regarded to be an important regulator molecule of immunity, infection and inflammation [9]. In particular, progranulin has a critical role in innate immunity against microorganisms. When progranulin knock-out mice were infected with *Listeria monocytogenes*, they were unable to clear bacteria effectively and displayed a greater inflammatory response as compared to the wild-type [11]. Progranulin-deficient mice were also highly sensitive to a lipopolysaccharide (LPS) challenge with increased susceptibility to lung injury and lung cell death after endotoxic shock [34]. Similar to our results, progranulin mRNA levels were shown to be upregulated along with TLR4 activation under inflammatory conditions in experimental animals [13]. Progranulin interacts with the TLR4 signaling pathway under hypoxic conditions [35], and TLR4 represents an important mammalian receptor for LPS [36]. Upregulated TLR4 and Myeloid Differentiation Primary Response 88 (MYD88) protein were demonstrated along with progranulin activation in our RT-qPCR-confirmed molecular network of the early antimicrobial response to sepsis. The TLR4-MYD88 signaling pathway is important in sepsis-induced ARDS, macrophage activation [37] and sepsis-associated myelosuppression [38]. Our model also identified massively upregulated sortilin (log_2_FC = 5.55) in blood cells. In neurons, where GRN haploinsufficiency plays a major role in frontotemporal lobar degeneration [39], sortilin has been identified as a high-affinity binding site of progranulin and is responsible for delivering progranulin to intracellular lysosomes for degradation [28]. Accordingly, SORT1 −/− mice showed strongly upregulated progranulin plasma levels [27]. This suggests that sortilin endocytosis may also determine circulating progranulin levels during sepsis.

Given the fact that increases of progranulin and procalcitonin in sepsis result from different tissues and different signaling pathways, one could assume that combining both biomarkers would increase overall diagnostic performance for detection of sepsis. Indeed, this seemed to be the case in a recent study in newborns with sepsis, where the combination with procalcitonin significantly improved the AUC value for progranulin alone from 0.786 to 0.806 [24]. However, this was not seen in our study in adults, where the AUC values for the combination of both biomarkers were well within the confidence intervals of the individual markers. These findings suggest a possible overlap of both signaling pathways in adults, which could result in downregulation of one of the biomarkers in response to upregulation of the other. In order to test this hypothesis, we performed additional pathway analyses and included the gene precursors of procalcitonin CALC1 and CALC2 into the antimicrobial signaling network, which had identified progranulin upregulation in early sepsis (Fig. 9). Increased plasma levels of CALC1 have been demonstrated in plasma of septic animals [28]. IPA analysis revealed upregulation of SORT1 by CALC1 (protein–protein interaction) and its gene product calcitonin [40]. Increased SORT1 expression may result in a lowering of progranulin levels by enhanced lysosomal degradation of progranulin [27]. The model suggests that increases of procalcitonin result in a decrease of progranulin concentrations, which might explain the finding that the combination of both biomarkers did not improve the overall diagnostic performance in our study.

## Conclusion

Progranulin has an important functional and regulatory role in early sepsis and is part of a molecular network activated during the early antimicrobial response to sepsis with sortilin (SORT1 gene) as an important co-regulator. Progranulin plasma concentrations are increased during the early stages of sepsis, are significantly increased in patients with pneumonia as a primary focus for sepsis and are also significantly higher in patients with confirmed COVID-19 pneumonia than in non-COVID-19 CAP. When using progranulin as a biomarker for sepsis and pneumonia, the diagnostic performance of this molecule is comparable to that of procalcitonin, and surpasses the other established biomarkers C-reactive protein and interleukin-6 with regard to sensitivity and specificity.

## Supplementary Information

**Additional file 1: Figure S1.** Comparison of C-reactive protein (CRP) (left graph, A) and interleukin-6 (IL-6) (right graph, B) plasma concentrations between healthy controls and patients with either a severe localized infection (e.g., a large peripheral abscess at high risk for sepsis), community-acquired pneumonia, sepsis or septic shock. Data are presented separately for the exploratory (blue boxplots) and the confirmatory study (orange boxplots). All symbols indicate *p* < 0.001; significant *p*-values ≥ 0.001 are given as numbers. # indicates a significant difference between patients with sepsis, septic shock or pneumonia when compared to healthy controls. + indicates a significant difference between patients with sepsis, pneumonia or healthy controls when compared to septic shock patients. **Figure S2.** Comparison of C-reactive protein (CRP) (left graph A) and interleukin-6 (IL-6) (right graph B) plasma concentrations according to sepsis of pulmonary, abdominal or other origin. Data are presented separately for the exploratory (blue boxplots) and the confirmatory study (orange boxplots). *Indicates *p* < 0.001; significant *p*-values ≥ 0.001 are given as numbers. **Figure S3.** Comparison of C-reactive protein (CRP) (left graph A) and interleukin-6 (IL-6) (right graph B) plasma concentrations between healthy controls; patients with SIRS and septic patients. Data are presented separately for the exploratory (blue boxplots) and the confirmatory study (orange boxplots). All symbols indicate *p* < 0.001; significant *p*-values ≥ 0.001 are given as numbers. **Figure S4.** ROC analysis for the differentiation between patients with localized infections against sepsis for progranulin in comparison to procalcitonin (PCT, A), C-reactive protein (CRP, B) and interleukin-6 (IL-6, C). The curves for progranulin are illustrated in red and the lines of the corresponding reference marker are outlined in blue (solid lines represent summary values from both cohorts). Lighter colors show measurements in the exploratory cohort and dashed lines show the corresponding measurements in the confirmatory (validation) sample. AUC values and statistical comparison for progranulin and procalcitonin are presented in the text and in Table S8 and S9 in more detail. **Figure S5.** Dot histograms illustrating the diagnostic performance of progranulin for the differentiation between COVID-19 (*n* = 22 = , community-acquired pneumonia (*n* = 28) and healthy individuals (*n* = 50). The lower row of graphs shows the absent diagnostic value of C-reactive protein, interleukin-6, procalcitonin and the leucocyte count in the study samples. Red lines indicate cut-off values. Outlier A in the progranulin histogram, presenting with lowest progranulin plasma concentrations in the healthy range (26.7 ng/ml) was mildly symptomatic for 14 days before hospital admission, was negatively tested for SARS-CoV-2 antibodies at admission and had only one positive nasal swap for SARS-CoV-19 at this time point; all three consecutive swaps were negative indicating low or no disease activity. A number of measurements were not available for interleukin-6 and procalcitonin. **Figure S6.** Flowchart representing the patient inclusion for sepsis, localized infection (infection) and community-acquired-pneumonia (pneumonia). The patients included in the SIRS group were scheduled for elective open-heart surgery. Patients were included depending on the availability of the investigating study team

## Data Availability

Additional data and materials are available from the corresponding author upon reasonable request.

## Abbreviations

ABCB1: ATP-binding cassette sub-family B member 1
AHNAK: Neuroblast differentiation-associated protein
ALOX5: Arachidonate 5-lipoxygenase
ANOVA: Analysis of variance
ARDS: Acute respiratory distress syndrome
AUC: Area under the curve
BCL2: B-cell lymphoma 2
BCL2L1: Bcl-2-like 1
BNIP2: BCL2/adenovirus E1B 19 kDa protein-interacting protein 2
BSG: Basigin
CALC1: Procalcitonin coding gene
CALC2: Calcitonin-related polypeptide beta
CAP: Community-acquired pneumonia
CAPG: Macrophage capping protein
CEBPB: CCAAT/enhancer-binding protein beta
CRP: C-reactive protein
ELISA: Enzyme-linked immunosorbent assay
FSCN1: Fascin actin-bundling protein 1
GRN: Progranulin coding gene
H3F3A: Histone H3.3
IL-10: Interleukin-10
IL1RN: Interleukin 1 receptor antagonist
IL-6: Interleukin-6
IPA: Ingenuity pathway analysis
LPS: Lipopolysaccharide
LTB: Lymphotoxin beta
MAN1A1: Mannosyl-oligosaccharide 1,2-alpha-mannosidase IA
miRNA: MicroRNA
mRNA: Messenger RNA
MYD88: Myeloid differentiation primary response 88
NCL: Nucleolin
NEDD4: Neural precursor cell expressed developmentally down-regulated protein 4
NGS: Next-generation sequencing
NT5E: 5′-Nucleotidase
PCT: Procalcitonin
PGRN: Progranulin
PPARG: Peroxisome proliferator-activated receptor gamma
PTEN: Phosphatase and tensin homolog
RARG: Retinoic acid receptor gamma
ROC: Receiver operating characteristic
RT-qPCR: Real-time quantitative polymerase chain reaction
SARS-CoV-2: Severe acute respiratory syndrome coronavirus type 2
SIRS: Systemic inflammatory response syndrome
SLC4A7: Solute carrier family 4, sodium bicarbonate cotransporter, member 7
SORT1: Sortilin coding gene
TGFBR1: Transforming growth factor beta receptor I
TLR4: Toll-like-receptor 4
TMBIM6: Transmembrane BAX inhibitor motif containing 6
TNF: Tumor necrosis factor
TP53: Tumor-protein 53
TYMS: Thymidylate synthetase
VEGFA: Vascular endothelial growth factor A

## Footnotes

1 No procalcitonin and C-reactive protein values were available for healthy controls.
