# Supplementary material for: Progranulin signaling in sepsis, community-acquired bacterial pneumonia and COVID-19: a comparative, observational study
Source: Intensive Care Med Exp. 2021 Sep 3;9:43. doi: 10.1186/s40635-021-00406-7 (PMC8412980; doi:10.1186/s40635-021-00406-7)
Supplement: Supplementary file 1 — Additional file 1: Figure S1. Comparison of C-reactive protein (CRP) (left graph, A) and interleukin-6 (IL-6) (right graph, B) plasma concentrations between healthy controls and patients with either a severe localized infection (e.g., a large peripheral abscess at high risk for sepsis), community-acquired pneumonia, sepsis or septic shock. Data are presented separately for the exploratory (blue boxplots) and the confirmatory study (orange boxplots). All symbols indicate p < 0.001; significant p-values ≥ 0.001 are given as numbers. # indicates a significant difference between patients with sepsis, septic shock or pneumonia when compared to healthy controls. + indicates a significant difference between patients with sepsis, pneumonia or healthy controls when compared to septic shock patients. Figure S2. Comparison of C-reactive protein (CRP) (left graph A) and interleukin-6 (IL-6) (right graph B) plasma concentrations according to sepsis of pulmonary, abdominal or other origin. Data are presented separately for the exploratory (blue boxplots) and the confirmatory study (orange boxplots). *Indicates p < 0.001; significant p-values ≥ 0.001 are given as numbers. Figure S3. Comparison of C-reactive protein (CRP) (left graph A) and interleukin-6 (IL-6) (right graph B) plasma concentrations between healthy controls; patients with SIRS and septic patients. Data are presented separately for the exploratory (blue boxplots) and the confirmatory study (orange boxplots). All symbols indicate p < 0.001; significant p-values ≥ 0.001 are given as numbers. Figure S4. ROC analysis for the differentiation between patients with localized infections against sepsis for progranulin in comparison to procalcitonin (PCT, A), C-reactive protein (CRP, B) and interleukin-6 (IL-6, C). The curves for progranulin are illustrated in red and the lines of the corresponding reference marker are outlined in blue (solid lines represent summary values from both cohorts). Lighter colors show measurements i [file 40635_2021_406_MOESM1_ESM.docx]

Progranulin Signaling in Sepsis, Community Acquired Bacterial Pneumonia and COVID-19: A Comparative, Observational Study

Florian Brandes^*1,3^, Melanie Borrmann^*1^, Dominik Buschmann^1/2^, Agnes S. Meidert^1^*,* Marlene Reithmair^3^, Markus Langkamp^4^, Lutz Pridzun^4^, Benedikt Kirchner^2^, Jean-Noël Billaud^5^, Nirav M. Amin^5^, Joseph C Pearson^5^, Matthias Klein^6^, Daniela Hauer^1^, Clarissa Gevargez Zoubalan^1^, Anja Lindemann^3^, Alexander Choukér^1^, Thomas W. Felbinger^7^, Ortrud K. Steinlein^3^, Michael W. Pfaffl^2^, Ines Kaufmann^7^, Gustav Schelling^1^

^1^Department of Anaesthesiology, University Hospital, Ludwig-Maximilians-University of Munich, Germany; ^2^Division of Animal Physiology and Immunology, TUM School of Life Sciences Weihenstephan, Technical University of Munich, Germany; ^3^Institute of Human Genetics, University Hospital, Ludwig-Maximilians-University, Munich, Germany; ^4^MEDIAGNOST Company, Aspenhausstr. 25, 72770 Reutlingen, Germany; ^5^QIAGEN Digital Insights, Redwood City, USA; ^6^Department of Neurology, University Hospital, Ludwig-Maximilians-University of Munich, Germany; ^7^Department of Anaesthesiology, Neuperlach Hospital, City Hospitals of Munich, Germany

*FB and MB should be considered joint first author.

**Additional online data**

**Corresponding author**

Florian Brandes

Department of Anaesthesiology

Campus Grosshadern

E-mail: florian.brandes@med.uni-muenchen.de

Phone: +4989440072743

Fax: +4989440078885

Content

[Additional Tables 3](#_Toc58927768)

[Additional Figures 14](#_Toc58927769)

[References for online additional files 20](#_Toc58927770)

# Additional Tables

**Table S1:** Comparison of demographic and clinical data between sepsis patients from the exploratory and the confirmatory study group measured at study inclusion (admittance to the ICU).

| **Parameter** | **Exploratory Cohort (n=114)** | **Confirmatory Cohort (n=127)** | **p -value** |
| --- | --- | --- | --- |
| Sex (m/f) | 67 / 47 | 78 / 49 | 0.774 |
| Age (years) | 61.5 (49.2-72.0) | 67.0 (57.0-77.0) | 0.003 |
| Size (cm) | 171.0 (165.0-180.0) | 172.5 (165.0-178.0) | 0.410 |
| Body mass index (kg/m^2^) | 26.7 (23.2-33.0) | 25.7 (22.1-29.6) | 0.061 |
| Weight (kg) | 81.0 (65.0-95.0) | 76.0 (65.5-92.5) | 0.067 |
| Duration of ICU theray (days) | 13.0 (7.0-20.8) | 13.0 (8.0-32.8) | 0.085 |
| Duration of hospital stay (days) | 26.0 (16.0-42.5) | 28.0 (15.5-44.0) | 0.252 |
| Duration of mechanical ventilation (days) | 7.0 (3.0-13.5) | 8.0 (2.0-21.0) | 0.274 |
| Severity of sepsis (sepsis / septic shock)^g^ | 39 / 75 | 34 / 93 | 0.265 |
| Death (yes / no) | 21 / 93 | 29 / 98 | 0.494 |
| ARDS (yes / no)^e^ | 46 / 68 | 43 / 84 | 0.363 |
| Use of hydrocortison (yes/no) | 100 / 14 | 95 / 32 | 0.017 |
| RIFLE score (failure / injury / no / risk)^a^ | 21 / 2 / 90 / 1 | 32 / 9 / 73 / 13 | <0.001 |
| AKIN score (no / stage I / stage II / stage III)^b^ | 84 / 2 / 1 / 27 | 73 / 13 / 9 / 32 | 0.002 |
| Pathogens (gram-negativ/gram-positiv/combined/viral/other/fungal/no pathogen detected) (n) | 27 / 30 / 0 / 7 / 16 / 5 / 29 | 26 / 38 / 2 / 6 / 16 / 3 / 36 | 0.63 |
| Localisation (pulmonary / abdominal / other) | 61 / 28 / 25 | 53 / 48 / 26 | 0.076 |
| Acute kidney injury (yes / no)^b^ | 47 / 67 | 63 / 64 | 0.24 |
| Renal replacement therapy (yes / no)^c^ | 41 / 73 | 40 / 87 | 0.551 |
| SAPS II score^d^ | 67.0 (54.2-78.8) | 49.0 (33.0-63.8) | <0.001 |
| APACHE II score^f^ | 29.0 (22.2-32.8) | 24.0 (17.0-31.0) | 0.002 |
| SOFA score^d^ | 13.0 (10.0-15.0) | 12.0 (8.0-14.0) | 0.003 |
| Maximal norepinephrine dose required (ug/kg/min) | 0.4 (0.2-0.7) | 0.3 (0.1-0.7) | 0.176 |
| Lactate (mmol/l) | 2.8 (1.8-5.5) | 2.6 (1.6-4.0) | 0.214 |
| Leukocyte count (G/l) | 11.5 (5.1-19.2) | 14.7 (10.0-21.0) | 0.021 |
| Progranulin (ng/ml) | 60.2 (44.5-89.4) | 56.0 (42.0-78.0) | 0.176 |
| Procalcitonin (ng/ml) | 4.2 (1.2-25.0) | 3.8 (1.1-12.8) | 0.238 |
| C-reactive protein (mg/dl) | 17.9 (10.6-28.0) | 21.8 (11.7-31.0) | 0.176 |
| Interleukin-6 (pg/ml) | 741.4 (261.6-2740.2) | 244.0 (85.2-1233.0) | <0.001 |

Data are given as median (IQR, interquartil range) or number per group, respectively. f: Acute Physiology And Chronic Health Evaluation score [1]

b: Acute Kidney Injury Network score [2] g: Sepsis was defined according to Sepsis-3 criteria [3]

c: The indication for renal replacement therapy was left to the discretion of the attending ICU physicians

d: Simplified Acute Physiology Score [4]

e: Indicates Acute Respiriatory Distress Syndrome, defined according to Berlin criteria [5]

**Table S2:** Demographic and clinical data from ICU patients after cardiac surgery with SIRS who were used as comparison group for patients from the exploratory and the confimatory group. Measurements were taken at day 0 after surgery while the patients underwent postoperative ICU treatment.

| **Parameter** | **SIRS comparison sample for exploratory study (n=90)** | **SIRS comparison sample for confirmatory study (n=92)** | **p-value** |
| --- | --- | --- | --- |
| Sex (m/f) | 68 / 22 | 70 / 22 | 0.929 |
| Age (years) | 67 (54-74) | 66 (58-75) | 0.143 |
| Body mass index (kg/m²)^a^ | 25.2 (23.6-28.8) | 28.1 (25.3-30.6) | <0.001 |
| Duration of cardiopulmonary bypass (min) | 120.0 (95.0-164.0) | 110.0 (87.5-159.0) | 0.179 |
| Duration of postoperative mechanical ventilation (hrs) | 16.0 (11.5-21.0) | 14.7 (11.3-19.3) | 0.127 |
| Rethoracotomy (yes/no) | 10 / 80 | 7 / 85 | 0.577 |
| Lowest PaO_2_ after surgery | 69.5 (63.8-74.2) | 77.0 (68.0-83.0) | 0.097 |
| Duration of hospital stay (d) | 15.0 (12.0-18.0) | 12.0 (10.0-15.0) | <0.001 |
| Progranulin after surgery (ng/ml) | 29.0 (26.0-35.0) | 28.0 (24.0-35.0) | 0.078 |
| Death after surgery (yes/no) | 4 / 86 | 1 / 91 | 0.351 |
| Procalcitonin after surgery (ng/ml) | 0.3 (0.1-0.6) | 0.1 (0.1-0.4) | 0.187 |
| C-reactive protein after surgery (mg/dl) | 7.4 (5.0-9.4) | 7.6 (5.5-9.7) | 0.309 |
| Interleukin-6 (pg/ml) | 268.9 (104.1-500.6) | 129.4 (77.1-187.2) | <0.001 |
| Lactate after surgery (mmol/L) | 3.0 (1.7-4.6) | 1.7 (1.1-3.2) | <0.001 |

All data are given as median (IQR, interquartil range) or number per group, respectively.

a: Body mass index

**Table S3:** Comparison of demographic and clinical data from patients with SIRS, localized infection or pneumonia with sepsis. Measurements were taken at admittance to the ICU for patients with SIRS and sepsis and at hospital admission for localized infection and pneumonia.

| **Parameter** | **SIRS (n=182)** | **p – value^a^** | **Localized Infection (n=48)** | **p-value^b^** | **Pneumonia^c^ (n=31)** | **p-value^d^** | **Sepsis (n=241)** |
| --- | --- | --- | --- | --- | --- | --- | --- |
| Sex (m/f) | 138 / 44 | 0.001 | 31 / 17 | 0.681 | 24 / 7 | 0.095 | 145 / 96 |
| Age | 66.0 (57.0-74.0) | 0.232 | 51.5 (35.8-64.0) | <0.001 | 73.0(61.0 - 81.0) | 0.008 | 64.0 (55.0-74.0) |
| Body mass index (kg/m^2^) | 27.3 (23.9-30.3) | 0.156 | 26.4 (23.6-28.7) | 0.416 | 24.9(23.1 - 30.5) | 0.347 | 26.2 (22.7-30.8) |
| Origin of infection (pulmonary /abdominal/ other) | -^e^ | - | 0 / 34 / 14 | <0.001 | 31 / 0 / 0 | <0.001 | 76/114/51 |
| Pathogens (gram-negativ/gram-positiv/combined/viral/fungal/other/no pathogen detected) (n) | -^e^ | - | 6 / 6 / 0 / 0 / 0 / 2 / 34 | <0.001 | 1 / 2 / 0 / 0 / 1 / 27 | <0.001 | 53 / 68 / 2 / 13 / 8 / 34 / 63 |
| Length of hospital stay (d) | -^f^ | - | 1 (1-1) | <0.001 | 9.0(5.0 - 11.0) | <0.001 | 10 (4-15) |
| Death (yes/no) | 5 / 177 | <0.001 | 0 / 48 | <0.001 | 0 / 31 | 0.01 | 50 / 191 |
| Progranulin (ng/ml) | 29.0 (25.0-35.0) | <0.001 | 29.0 (24.7-36.0) | <0.001 | 38.0 (33.5–41.0) | <0.001 | 57.5 (42.8-84.9) |
| Procalcitonin (ng/ml) | 0.1 (0.1-0.5) | <0.001 | 0.3 (0.2-0.4) | <0.001 | 0.2 (0.1–0.7) | <0.001 | 4.1 (1.1-19.3) |
| C-reactive protein (mg/dl) | 7.4 (5.2-9.6) | <0.001 | 1.8 (0.9-5.5) | <0.001 | 11.8 (4.8-18.4) | <0.001 | 20.2 (10.8-29.8) |
| Interleukin-6 (pg/ml) | 167.2 (90.1-387.4) | <0.001 | 173.0 (93.5-323.0) | <0.001 | 19.1(0.0-39.2) | <0.001 | 512.4 (127.0-1889.4) |

Data are given as median (IQR, interquartil range) or number per group, respectively.
a: p-value between SIRS and sepsis.
b: p-value between localized infection and sepsis.
c: Total sample.
d: p-value between pneumonia and sepsis.
e: No comparision, because SIRS patient cohort per definition has a sterile inflamation.
f: No comparision was made, because SIRS patients length of stay is due to OP trauma and not SIRS.

**Table S4:** Demographic and clinical data of the ICU patients who survived and patients who died during ICU therapy.

| **Parameter** | **Non-survivor (n=50)** | **Survivor (n=191)** | **p-value** |
| --- | --- | --- | --- |
| Sex (m/f) | 31 / 19 | 114 / 77 | 0.892 |
| Age (years) | 69.0 (58.2-77.5) | 63.0 (51.0-73.0) | 0.009 |
| Body mass index (kg/m^2^) | 24.3 (21.8-29.4) | 26.4 (23.1-31.3) | 0.050 |
| Weight (kg) | 75.0 (61.1-90.0) | 80.0 (68.0-98.0) | 0.052 |
| Duration of ICU theray (days) | 11.5 (4.8-24.2) | 13.0 (8.0-25.0) | 0.142 |
| Duration of hospital stay (days) | 19.5 (8.5-34.5) | 29.0 (17.0-45.0) | 0.003 |
| Duration of mechanical ventilation (days) | 7.0 (3.0-17.5) | 8.5 (2.0-18.0) | 0.480 |
| Severity of sepsis (sepsis / septic shock)^g^ | 4 / 46 | 69 / 122 | <0.001 |
| ARDS (yes / no)^e^ | 21 / 29 | 68 / 123 | 0.503 |
| Use of hydrocortison (yes/no) | 46 / 4 | 149 / 42 | 0.041 |
| RIFLE score (failure / injury / no / risk)^a^ | 24 / 3 / 21 / 2 | 29 / 8 / 142 / 12 | <0.001 |
| AKIN score (no / stage I / stage II / stage III)^b^ | 19 / 2 / 3 / 26 | 138 / 13 / 7 / 33 | <0.001 |
| Pathogens (gram-negativ/gram-positiv/combined/viral/other/fungal/ no pathogen detected) (n) | 12 / 19 / 1 / 0 / 3 / 5 / 10 | 41 / 49 / 1 / 13 / 31 / 3 / 53 | 0.003 |
| Localisation (abdominal / other / pulmonary) | 19 / 13 / 18 | 57 / 38 / 96 | 0.198 |
| Acute kidney injury (yes / no)^b^ | 41 / 9 | 69 / 122 | <0.001 |
| Renal replacement therapy (yes / no)^c^ | 35 / 15 | 46 / 145 | <0.001 |
| SAPS II score^d^ | 66.0 (49.0-80.0) | 58.0 (40.5-70.0) | 0.003 |
| APACHE II score^f^ | 30.0 (25.0-33.0) | 25.0 (18.0-31.0) | 0.002 |
| SOFA score^d^ | 14.0 (11.8-16.0) | 12.0 (9.0-14.0) | <0.001 |
| Maximal norepinephrine dose required (ug/kg/min) | 0.7 (0.6–1.0) | 0.3 (0.1–0.6) | <0.001 |
| Lactate (mmol/l) | 4.2 (2.8-10.4) | 2.3 (1.5-3.9) | <0.001 |
| Leukocyte count (G/l) | 14.8 (6.5-20.4) | 13.3 (8.5-20.4) | 0.412 |
| Progranulin (ng/ml) | 73.7 (50.4-104.5) | 55.7 (41.0-76.2) | 0.004 |
| Procalcitonin (ng/ml) | 4.3 (2.6-13.1) | 3.9 (1.0-19.8) | 0.125 |
| C-reactive protein (mg/dl) | 18.9 (7.9-29.9) | 20.2 (12.0-29.6) | 0.186 |
| Interleukin-6 (pg/ml) | 403.7 (138.2–7169.0) | 522.5 (127.0–1642.1) | 0.265 |

Data are combined from both the exploratory and the confirmatory group. Data are given as median (IQR, interquartil range) or number per group, respectively. Disease severity scores (SOFA, APACHE II and SAPS II) were calculated and biochemical measurements performed at admittance to the ICU.
a:^:^ Risk, Injury, Failure, Loss of Kidney Function, and End-stage Kidney Disease score

b: Acute Kidney Injury Network score

c: The indication for renal replacement therapy was left to the discretion of the attending ICU physicians

d: Simplified Acute Physiology Score
e: Indicates Acute Respiriatory Distress Syndrome, defined according to Berlin criteria

f: Acute Physiology And Chronic Health Evaluation score  g: Sepsis was defined according to Sepsis-3 criteria

**Table S5:** Demographic and clinical data from the healthy volunteers compared to patients with sepsis.

| **Parameter** | **Volunteers (n=53)** | **Sepsis (n=241)** | **p-value** |
| --- | --- | --- | --- |
| Sex (m/f) | 28 / 25 | 145 / 96 | 0.407 |
| Age (years) | 51.0 (41.0-53.8) | 64.0 (55.0-74.0) | <0.001 |
| Body mass index (kg/m²)^a^ | 24.9 (23.5-26.0) | 26.2 (22.7-30.8) | 0.019 |
| Progranulin (ng/ml) | 28.7 (25.5-31.7) | 57.5 (42.8-84.9) | <0.001 |
| Interleukin-6 (pg/ml) | 26.0 (3.0-50.0) | 512.4 (127.0-1889.4) | <0.001 |

Data are given as median (IQR, interquartil range) or number per group, respectivly.

**Table S6**: Comparison of demographic and clinical data between patients selected and not selected for high-throughput analysis from the confirmatory cohort.

| **Parameter** | **Non-NGS (n=235)** | **NGS (n=7)** | **p-value** |
| --- | --- | --- | --- |
| Sex (m/f) | 140 / 95 | 6 / 1 | 0.317 |
| Age (years) | 65.0(55.0 - 74.0) | 58.0(54.0 - 58.0) | 0.030 |
| Body mass index (kg/m^2^) | 26.2(22.6 - 30.9) | 28.3(24.7 - 29.9) | 0.243 |
| Weight (kg) | 80.0(65.0 - 95.0) | 98.0(72.5 - 100.0) | 0.165 |
| Duration of ICU theray (days) | 13.0(7.0 - 23.8) | 25.0(18.0 - 36.5) | 0.019 |
| Duration of hospital stay (days) | 27.5(15.8 - 44.0) | 30.0(22.0 - 51.5) | 0.190 |
| Duration of mechanical ventilation (days) | 7.5(2.0 - 17.5) | 16.0(11.2 - 23.0) | 0.048 |
| Severity of sepsis (sepsis / septic shock)^g^ | 73 / 162 | 0 / 7 | 0.178 |
| Death (yes / no) | 46 / 189 | 4 / 3 | 0.052 |
| ARDS (yes / no)^e^ | 86 / 149 | 4 / 3 | 0.477 |
| Use of hydrocortison (yes/no) | 189 / 46 | 7 / 0 | 0.417 |
| RIFLE score (failure / injury / no / risk)^b^ | 51 / 11 / 160 / 13 | 2 / 0 / 4 / 1 | 0.684 |
| AKIN score (no / stage I / stage II / stage IIIc)^b^ | 154 / 14 / 10 / 57 | 4 / 1 / 0 / 2 | 0.757 |
| Pathogens (gram-negativ/gram-positiv/combined/viral/other/fungal/no pathogen detected) (n) | 51 / 64 / 2/ 13 / 34 / 8 /63 | 2 / 5 / 0 / 0 / 0 / 0 / 0 | 0.300 |
| Localisation (pulmonary / abdominal / other) | 112 / 73 / 50 | 3 / 3 / 1 | 0.782 |
| Acute kidney injury (yes / no)^b^ | 107 / 128 | 3 / 4 | 0.806 |
| Renal replacement therapy (yes / no)^c^ | 77 / 158 | 4 / 3 | 0.347 |
| SAPS II score^d^ | 60.0(43.5 - 72.5) | 65.5(58.2 - 67.5) | 0.297 |
| APACHE II score^f^ | 26.0(19.0 - 32.0) | 28.0(21.5 - 32.5) | 0.399 |
| SOFA score^d^ | 12.0(9.0 - 14.5) | 16.0(13.0 - 16.0) | 0.023 |
| Maximal norepinephrine dose required (ug/kg/min) | 0.3(0.1 - 0.7) | 0.5(0.3 - 0.8) | 0.162 |
| Lactate (mmol/l) | 2.5 (1.6–4.4) | 4.3(3.5 - 11.9) | 0.020 |
| Leukocyte count (G/l) | 13.3(8.1 - 20.2) | 17.5(8.6 - 23.7) | 0.298 |
| Prograulin (ng/ml) | 57.0(42.4 - 85.0) | 58.1 (43.5–86.1) | 0.270 |
| Procalcitonin (ng/ml) | 4.1(1.1 - 19.2) | 2.5(2.0 - 7.7) | 0.384 |
| C-reactive protein (mg/dl) | 20.6(11.5 - 29.9) | 8.7(3.5 - 12.2) | 0.012 |
| Interleukin -6 (pg/ml) | 512.4(127.0 - 1934.5) | 370.0(193.0 - 766.0) | 0.443 |

**Table S7:** Comparison of demographic and clinical data between patients selected and not selected for RT-qPCR confirmation from the confirmatory cohort.

| **Parameter** | **qPCR (n=40)** | **Non-qPCR (n=201)** | **p-value** |
| --- | --- | --- | --- |
| Sex (m/f) | 25 / 15 | 120 / 81 | 0.878 |
| Age (years) | 66.5(56.8 - 78.0) | 63.0(54.0-73.0) | 0.175 |
| Body mass index (kg/m^2^) | 25.2(22.2 - 31.0) | 26.3 (22.9-30.7) | 0.293 |
| Weight (kg) | 77.0(68.0 - 100.0) | 80.0 (65.0-95.0) | 0.403 |
| Duration of ICU theray (days) | 12.0(9.0 - 28.0) | 13.0 (7.0-24.0) | 0.488 |
| Duration of hospital stay (days) | 29.0 (13.2-38.8) | 27.0(16.0 - 45.0) | 0.491 |
| Duration of mechanical ventilation (days) | 9.5(3.5 - 21.0) | 7.0 (2.0-16.0) | 0.095 |
| Severity of sepsis (sepsis / septic shock)^g^ | 8 / 32 | 65 / 136 | 0.173 |
| Death (yes / no) | 9 / 31 | 41 / 160 | 0.932 |
| ARDS (yes / no)^e^ | 14 / 26 | 75 / 126 | 0.922 |
| Use of hydrocortison (yes/no) | 36 / 4 | 159 / 42 | 0.167 |
| RIFLE score (failure / injury / no / risk)^b^ | 12 / 4 / 19 / 5 | 41 / 7 / 144 / 9 | 0.011 |
| AKIN score (no / stage I / stage II / stage IIIc)^b^ | 19 / 5 / 4 / 12 | 138 / 10 / 6 / 47 | 0.020 |
| Pathogens (gram-negativ/gram-positiv/combined/viral/other/fungal/no pathogen detected) (n) | 9 / 12 / 1 / 0 / 4 / 0 / 14 | 44 / 56 / 1 / 13 / 30 / 8 / 49 | 0.183 |
| Localisation (pulmonary / abdominal / other) | 20 / 15 / 5 | 94 / 61 / 46 | 0.314 |
| Acute kidney injury (yes / no)^b^ | 23 / 17 | 87 / 114 | 0.140 |
| Renal replacement therapy (yes / no)^c^ | 14 / 26 | 67 / 134 | 0.984 |
| SAPS II score^d^ | 48.5(30.8 - 60.0) | 62.0(46.5 - 74.0) | <0.001 |
| APACHE II score^f^ | 23.0 (19.0–31.0) | 27.0 (19.0–32.0) | 0.102 |
| SOFA score^d^ | 12.0 (9.0–13.0) | 13.0 (10.0–15.0) | 0.067 |
| Maximal norepinephrine dose required (ug/kg/min) | 0.3 (0.1–0.6) | 0.4 (0.2–0.7) | 0.228 |
| Lactate (mmol/l) | 2.7 (1.6–4.3) | 2.5 (1.7–4.8) | 0.468 |
| Leukocyte count (G/l) | 14.4(8.2 - 18.5) | 13.8(8.0 - 20.9) | 0.459 |
| Prograulin (ng/ml) | 52.5(40.6 - 76.0) | 58.0(43.3 - 85.7) | 0.187 |
| Procalcitonin (ng/ml) | 4.7(1.4 - 14.7) | 3.9(1.1 - 20.5) | 0.440 |
| C-reactive protein (mg/dl) | 22.0(14.6 - 33.0) | 18.8(10.4 - 28.8) | 0.096 |
| Interleukin -6 (pg/ml) | 230.5(76.0 - 1585.5) | 565.5(139.8 - 1983.9) | 0.082 |

Data are given as median (IQR, interquartil range) or number per group, respectively f: Acute Physiology And Chronic Health Evaluation score

a: Risk, Injury, Failure, Loss of Kidney Function, and End-stage Kidney Disease score (3) g: Sepsis was defined according to Sepsis-3 criteria

b: Acute Kidney Injury Network score

c. The indication for renal replacement therapy was left to the discretion of the attending ICU physicians

d. Simplified Acute Physiology Score

e: Indicates Acute Respiriatory Distress Syndrome, defined according to Berlin criteria

**Table S8:** Comparison of AUC values between progranulin, procalcitonin, interleukin-6 and C-reactive protein in all subgroups. Values in parentheses behind AUC numbers are 95% confidence intervals. P-values were calculated using the paired deLong test [6]. All p-values were calculated for the comparison of AUC values to those of progranulin values.

| **Subgroup** | **Sample** | **Progranulin** | **Procalcitonin** | **p-value** | **Interleukin-6** | **p-value** | **C-reactive protein** | **p-value** |
| --- | --- | --- | --- | --- | --- | --- | --- | --- |
| Sepsis (n=114) vs. healthy controls (n=32) | Exploratory (n=146) | 0.95 (0.91-0.98) | -^a^ | - | -^a^ | - | -^a^ | - |
| Sepsis (n=127) vs. healthy controls (n=21) | Confirmatory (n=148) | 0.91 (0.85-0.96) | -^a^ | - | -^a^ | - | -^a^ | - |
| Sepsis (n=241) vs. Healthy Controls (n=53) | Combined (n=294) | 0.93 (0.92-0.96) | -^a^ | - | -^a^ | - | -^a^ | - |
| Sepsis (n=114) vs. SIRS^b^ (n=90) | Exploratory (n=204) | 0.91 (0.87-0.95) | 0.93 (0.86-0.97) | 0.551 | 0.73 (0.65-0.81) | <0.001 | 0.85 (0.80-0.91) | 0.061 |
| Sepsis (n=127) vs. SIRS^b^ (n=92) | Confirmatory (n=219) | 0.90 (0.85-0.94) | 0.91 (0.86-0.96) | 0.656 | 0.64 (0.56-0.73) | <0.001 | 0.83 (0.77-0.89) | 0.051 |
| Sepsis (n=241) vs SIRS^b^ (n=182) | Combined (n=423) | 0.90 (0.87-0.93) | 0.92 (0.88-0.96) | 0.323 | 0.68 (0.62-0.73) | <0.001 | 0.84 (0.8-0.88) | 0.009 |
| Sepsis (n=114) vs. loc. Infection^c^(n=24) | Exploratory (n=138) | 0.92 (0.87-0.97) | 0.93 (0.87-0.97) | 0.846 | 0.75 (0.65-0.85) | 0.001 | 0.83 (0.68-0.95) | 0.140 |
| Sepsis (n=127) vs. loc. infection^c^ (n=24) | Confirmatory (n=151) | 0.88 (0.81-0.93) | 0.84 (0.73-0.94) | 0.280 | 0.57 (0.44-0.72) | <0.001 | 0.88 (0.76-0.98) | 0.900 |
| Sepsis (n=241) vs. loc. infection^c^ (n=48) | Combined (n=289) | 0.90 (0.86-0.94) | 0.89 (0.84-0.94) | 0.790 | 0.68 (0.60-0.75) | <0.001 | 0.86 (0.78-0.94) | 0.334 |
| Sepsis (n=127) vs. non-COVID-19 pneumonia^d^ (n=31) | Confirmatory (n=158) | 0.77 (0.68-0.85) | 0.83 (0.73-0.92) | 0.36 | 0.86 (0.77-0.93) | 0.06 | 0.69 (0.58-0.79) | 0.22 |
| Non-COVID-19 pneumonia (n=31) vs. COVID-19 pneumonia (n=22) | Combined (n=53) | 0.91 (0.8-1.0) | 0.79 (0.66-0.92)^e^ | <0.001 | 0.69 (0.5-0.86) | 0.008 | 0.70 (0.59-0.84) ^e^ | <0.001 |
| Pneumonia (n=31) vs healthy controls (n=53) | Combined (n=84) | 0.87 (0.77-0.94) | -^a^ | -- | -^a^ | - | -^a^ | - |
| Death (n=21) by sepsis (n=114) | Exploratory (n=114) | 0.60 (0.47-0.74) | 0.53 (0.41-0.65) | 0.404 | 0.56 (0.42-0.71) | 0.717 | 0.35 (0.2-0.52) | 0.006 |
| Death (n=29) by sepsis (n=127) | Confirmatory (n=127) | 0.66 (0.53-0.78) | 0.60 (0.46-0.73) | 0.265 | 0.52 (0.35-0.66) | 0.072 | 0.52 (0.4-0.65) | 0.110 |
| Death (n=50) by sepsis (n=241) | Combined (n=241) | 0.63 (0.54-0.72) | 0.56 (0.46-0.66) | 0.143 | 0.53 (0.42-0.64) | 0.145 | 0.46 (0.35-0.55) | 0.006 |

a: C-reactive protein, Procalcitonin and Interleukin-6 measurements were not available in healthy controls.

b:Indicates systemic inflammatory response in patients after cardiac surgery in the absence of sepsis.

c: Patients with localized infections presented with large peripheral abscesses or disseminated skin infections at risk for sepsis.

d: Patients admitted to the emergency room of Munich University Hospital with suspected community acquired pneumonia (CAP). CAP was defined as an acute infection of the pulmonary parenchyma in a patient who has acquired the infection in the community and has not had recent hospitalization or association with other healthcare facilities such as nursing homes, dialysis centers, and outpatient clinics. Patients with malignancies, severe comorbid metabolic or cardiovascular disorders or after transplantation were excluded. We summarized CAP patients of both groups and compared them to the confirmatory sepsis and septic shock patients.
e: AUC was inverted due to the fact, that procalcitonin and c-reactive protein negatively predicted the possiblilty of a viral pneumonia

**Table S9:** Cut-off values of progranulin, procalcitonin, C-reactive protein and interleukin-6. Sensitivity and specificity values were calculated according to Youden et al. [7] using an iteration until a minmum sensitivity of at least 80% was reached and the best specificity was then calculated accordingly. Values in parentheses after cut-offs give sensitivity followed by specifity.

| **Subgroup** | **Sample Cohort** | **Progranulin (ng/ml)** | **Procalcitonin (ng/ml)** | **Interleukin-6 (pg/ml)** | **C-reactive protein (mg/dl)** |
| --- | --- | --- | --- | --- | --- |
| Sepsis (n=114) vs healthy controls (n=32) | Exploratory (n=146) | 34.60 (91.2% / 93.5%) | -^a^ | -^a^ | -^a^ |
| Sepsis (n=127) vs healthy controls (n=21) | Confirmatory (n=148) | 35.00 (84.6% / 90.0%) | -^a^ | -^a^ | -^a^ |
| Sepsis (n=241) vs healthy controls^a^ (n=53) | Combined (n=294) | 34.62 (87.8% / 92.2%) | -^a^ | -^a^ | -^a^ |
| Sepsis (n=114) vs SIRS^b^ (n=90) | Exploratory (n=204) | 38.44 (87.6% / 85.4%) | 1.00 (81.9% / 100.0%) | 204.47 (80.2% / 42.2%) | 9.60 (81.4% / 77.1%) |
| Sepsis (n=127) vs SIRS^b^ (n=92) | Confirmatory (n=219) | 38.00 (81.2% / 81.5%) | 0.79 (80.2% / 90.0%) | 95.86 (70,3% / 36,21%) | 9.12 (80.4% / 68.2%) |
| Sepsis (n=241) vs SIRS^b^ (n=182) | Combined (n=423) | 38.44 (83.5% / 85.7%) | 0.80 (81.6% / 92.0%) | 88.00 (80.2% / 24.8%) | 9.60 (80.4% / 74.2%) |
| Sepsis (n=114) vs. loc. infection^c^ (n=24) | Exploratory (n=138) | 38.44 (87.6% / 87.5%) | 0.70 (86.2% / 95.0%) | 204.47 (80.2% / 54.2%) | 5.50 (93.1% / 68.8%) |
| Sepsis (n=127) vs. loc. infection^c^ (n=24) | Confirmatory (n=151) | 35.00 (84.6% / 69.6%) | 0.69 (81.3% / 71.4%) | 13.00 (92.1% / 9.1%) | 3.20 (95.5% / 81.0%) |
| Sepsis (n=241) vs. loc. infection^c^ (n=48) | Combined (n=289) | 39.33 (80.9% / 87.2%) | 0.69 (83.8% / 88.9%) | 25.20 (88.2% / 20.0%) | 5.50 (90.7% / 75.7%) |
| Sepsis (n=127) vs. non-COVID-19 pneumonia^d^ (n=31) | Confirmatory (n=158) | 38.0 (81.2% / 45.2%) | 0.79 (80.2% / 80.0%) | 51.0 (81.2% / 80.0%) | 6.32 (88.4% / 36.7%) |
| Non-COVID-19 pneumonia (n=31) vs. COVID-19 pneumonia (n=22) | Combined (n=53) | 49.65 (90.9% / 93.5%) | 0.0 (100.0% / 0.0%) | 4.6 (100.0% / 33.3%) | 0.2 (100.0% / 0.0%) |
| Pneumonia^d^ (n=31) vs healthy controls (n=53) | Combined (n=84) | 33.00 (83.9% / 82.4%) | -^a^ | -^a^ | -^a^ |
| Death (n=21) by sepsis (n=114) | Exploratory (n=114) | 43.12 (95.0% / 26.9%) | 1.90 (93.8% / 37.2%) | 294.96 (81.0% / 28.9%) | 2.90 (100.0% / 2.4%) |
| Death (n=29) by sepsis (n=127) | Confirmatory (n=127) | 49.71 (80.8% / 44.0%) | 2.15 (84.2% / 37.5%) | 30.30 (95.2% / 15.0%) | 3.20 (100.0% / 6.0%) |
| Death (n=50) by sepsis (n=241) | Combined (n=241) | 47.02 (80.4% / 35.3%) | 2.10 (85.7% / 39.3%) | 30.30 (90.5% / 13.5%) | 2.90 (100.0% / 4.2%) |

a:C-reactive protein, Procalcitonin and Interleukin-6 measurements were not available in healthy controls.

b: Indicates systemic inflammatory response in patients after cardiac surgery in the absence of sepsis.

c: Patients treated in the ICU with localized infections presented with large peripheral abscesses or disseminated skin infections at risk for sepsis.

d: Patients admitted to the emergency room of Munich University Hospital with suspected community acquired pneumonia (CAP).

CAP was defined as an acute infection of the pulmonary parenchyma in a patient who has acquired the infection in the community and has not had recent hospitalization or association with other healthcare facilities such as nursing homes, dialysis centers, and outpatient clinics. Patients with malignancies, severe comorbid metabolic or cardiovascular disorders or transplantation were excluded. We combined CAP patients from both groups and compared them to the confirmatory sepsis patients.

**Table S10:** Comparison of demographic and clinical data between patients with a confirmed SARS-CoV-2 infection associated pneumonia vs. patients presenting with non-COVID-19 pneumonia. The COVID-19 group consisted of patients with SARS-CoV-2-pneumonia recruited soon after admittance to the University Hospital of Munich (LMU) isolation facility. All COVID-19 patients were recruited between 03/16/2020 and 05/04/20 and had at least one positive nasal swap for the novel SARS-CoV-19 virus (SARS-CoV-2-RNA PCR test, RdRP-Gen IP4) with typical symptoms.

| **Parameter** | **COVID-19 pneumonia (n=22)** | **Bacterial pneumonia (n=31)** | **p-value** |
| --- | --- | --- | --- |
| Sex (m/f) | 19 / 3 | 24 / 7 | 0.643 |
| Age (years) | 64.5 (55.0 - 75.0) | 73.0 (61.0 - 81.0) | 0.088 |
| Body mass index (kg/m^2^) | 26.6 (24.5 - 30.8) | 24.9 (23.1 - 30.5) | 0.117 |
| Weight (kg) | 86.0 (75.0 - 110.0) | 80.0 (70.0 - 89.0) | 0.079 |
| Duration of hospital stay (days) | 16.0 (11.0 - 29.0) | 9.0 (5.0 - 11.0) | 0.001 |
| Death (yes / no) | 3 / 19 | 0 / 31 | 0.130 |
| ARDS^a^ (yes / no) | 9 / 13 | 0 / 31 | <0.001 |
| Pathogens (gram-negativ/gram-positiv/combined/viral/other/fungal/no pathogen detected) (n) | 0 / 0 / 0 / 22 / 0 / 0 / 0 | 1 / 2 / 0 / 0 / 1 / 0 / 27 | <0.001 |
| Leukocyte count (G/l) | 5.0 (3.1 - 7.9) | 9.4 (7.0 - 12.4) | <0.001 |
| Prograulin (ng/ml) | 67.6 (56.6 - 96.0) | 38.0 (33.5 - 41.0) | <0.001 |
| Procalcitonin (ng/ml) | 0.0 (0.0 - 0.2) | 0.2 (0.1 - 0.7) | <0.001 |
| C-reactive protein (mg/dl) | 6.2 (2.1 - 10.8) | 11.8 (4.8 - 18.4) | 0.007 |
| Interleukin -6 (pg/ml) | 55.3 (17.6 - 94.6) | 19.1 (0.0 - 39.2) | 0.028 |

Data are given as median (IQR, interquartil range) or number per group, respectively

^a^ Indicates Acute Respiriatory Distress Syndrome, defined according to Berlin criteria

**Table S11:** Comparison of demographic and clinical data between patients with a community acquired pneumonia, with and without the necessity of ICU treatment.

| **Parameter** | **Moved to ICU (n=10)** | **No ICU therapy necessary (n=43)** | **p-value** |
| --- | --- | --- | --- |
| Sex (m/f) | 9 / 1 | 34 / 9 | 0.729 |
| Age (years) | 67.0(63.0 - 74.5) | 73.0(59.0 - 80.0) | 0.265 |
| Body mass index (kg/m^2^) | 26.1(24.5 - 27.3) | 25.2(23.7 - 30.8) | 0.433 |
| Weight (kg) | 80.0(70.0 - 90.0) | 84.0(72.0 - 94.0) | 0.488 |
| Duration of hospital stay (days) | 29.0(21.0 - 31.0) | 10.0(7.0 - 14.0) | 0.001 |
| Death (yes / no) | 3 / 7 | 0 / 43 | 0.003 |
| ARDS^a^ (yes / no) | 9 / 1 | 0 / 43 | <0.001 |
| Pathogens (gram-negativ/gram-positiv/combined/viral/other/fungal/no pathogen detected) (n) | 0 / 0 / 0 / 10 / 0 / 0 / 0 | 1 / 2 / 0 / 12 / 1 / 0 / 27 | 0.002 |
| Leukocyte count (G/l) | 6.6(4.6 - 7.8) | 8.4(5.7 - 12.0) | 0.034 |
| Prograulin (ng/ml) | 91.5(58.3 - 118.1) | 41.0(35.0 - 52.0) | <0.001 |
| Procalcitonin (ng/ml) | 0.0 (0.0 - 0.2) | 0.2(0.0 - 0.3) | 0.083 |
| C-reactive protein (mg/dl) | 10.0(6.4 - 10.8) | 9.0(2.3 - 16.2) | 0.495 |
| Interleukin -6 (pg/ml) | 106.2(70.7 - 178.5) | 18.4(6.6 - 39.2) | <0.001 |

Data are given as median (IQR, interquartil range) or number per group, respectively

^a^ Indicates Acute Respiriatory Distress Syndrome, defined according to Berlin criteria

**Table S12:** Contingency table representing progranulin as an index test to differentiate between study groups.

The threshold used was identified by using an optimized Youden index as explained and shown in Table 9.

|  | Differentiation between | | Differentiation between | | Differentiation between | |
| --- | --- | --- | --- | --- | --- | --- |
|  | **Volunteer** | **Sepsis** | **SIRS** | **Sepsis** | **Localized infection** | **Sepsis** |
| **PGRN above theshold** | 3 | 194 | 22 | 192 | 6 | 186 |
| **PGRN below threshold** | 48 | 36 | 132 | 38 | 41 | 44 |
|  | Differentiation between | | Differentiation between | | Differentiation between | |
|  | **non-COVID-19 CAP** | **COVID-19** | **Volunteer** | **non-COVID-19 CAP** | **Survivor** | **Death** |
| **PGRN above theshold** | 2 | 20 | 9 | 26 | 119 | 36 |
| **PGRN below threshold** | 29 | 2 | 42 | 5 | 65 | 10 |

Due to missing PGRN values in subgroups the total number of cases may not sum up to the total study population.

# Additional Figures


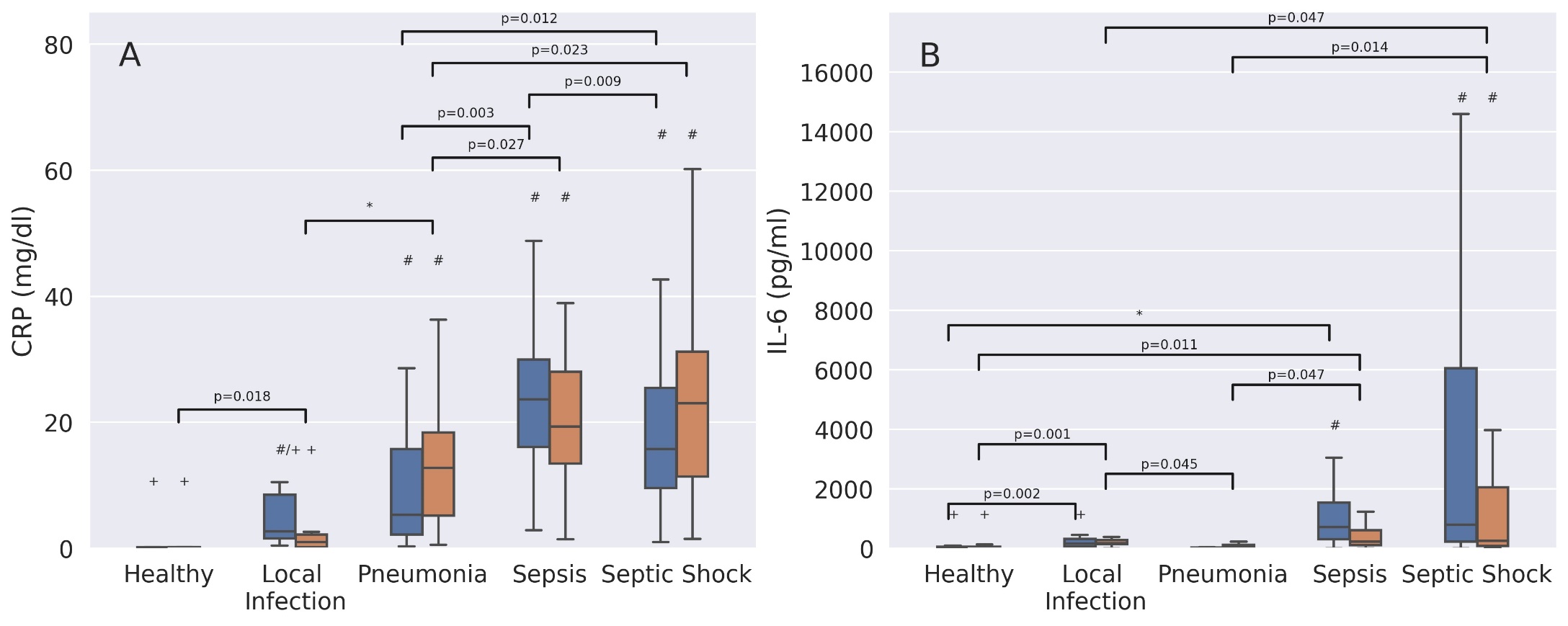


**Figure S1:** Comparison of C-reactive protein (CRP) (left graph, A) and interleukin-6 (IL-6) (right graph, B) plasma concentrations between healthy controls and patients with either a severe localized infection (e.g. a large peripheral abscess at high risk for sepsis), community acquired pneumonia, sepsis or septic shock. Data are presented separately for the exploratory (blue boxplots) and the confirmatory study (orange boxplots). All symbols indicate p<0.001; significant p-values ≥0.001 are given as numbers. # indicates a significant difference between patients with sepsis, septic shock or pneumonia when compared to healthy controls. + indicates a significant difference between patients with sepsis, pneumonia or healthy controls when compared to septic shock patients.


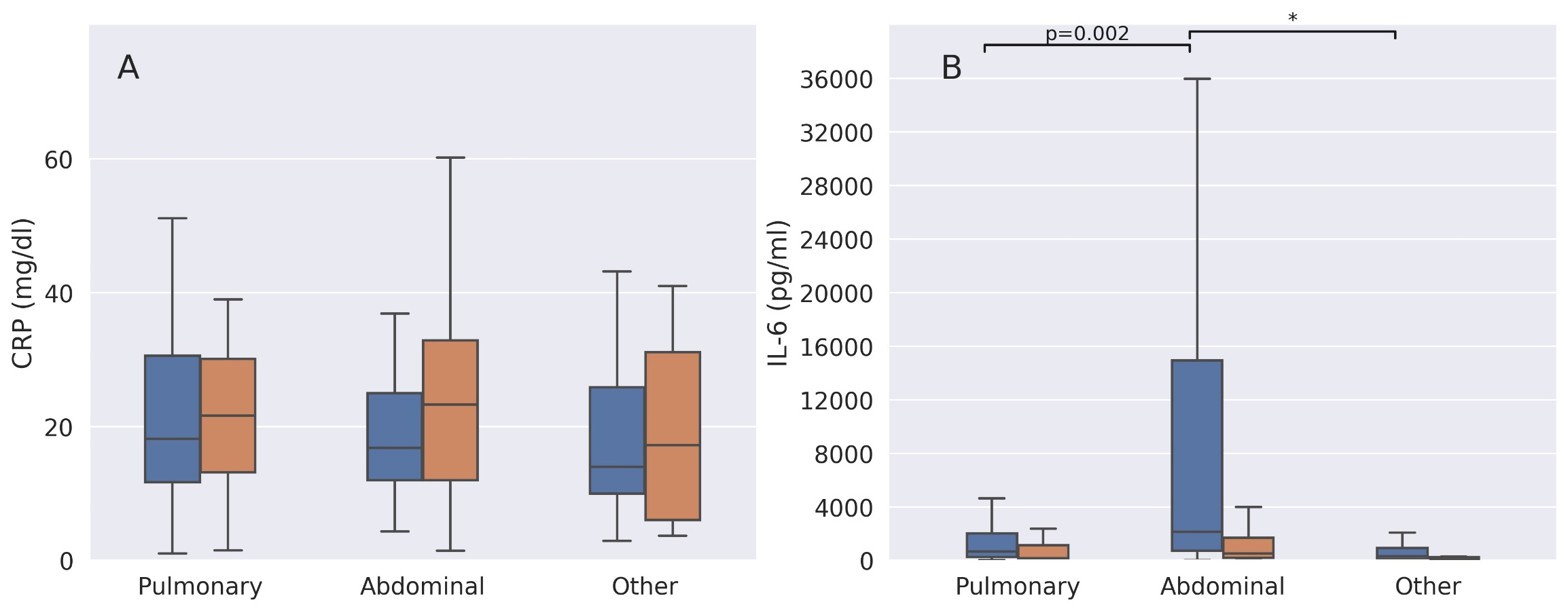


**Figure S2:** Comparison of C-reactive protein (CRP) (left graph A) and interleukin-6 (IL-6) (right graph B) plasma concentrations according to sepsis of pulmonary, abdominal or other origin. Data are presented separately for the exploratory (blue boxplots) and the confirmatory study (orange boxplots). * indicates p<0.001; significant p-values ≥0.001 are given as numbers.


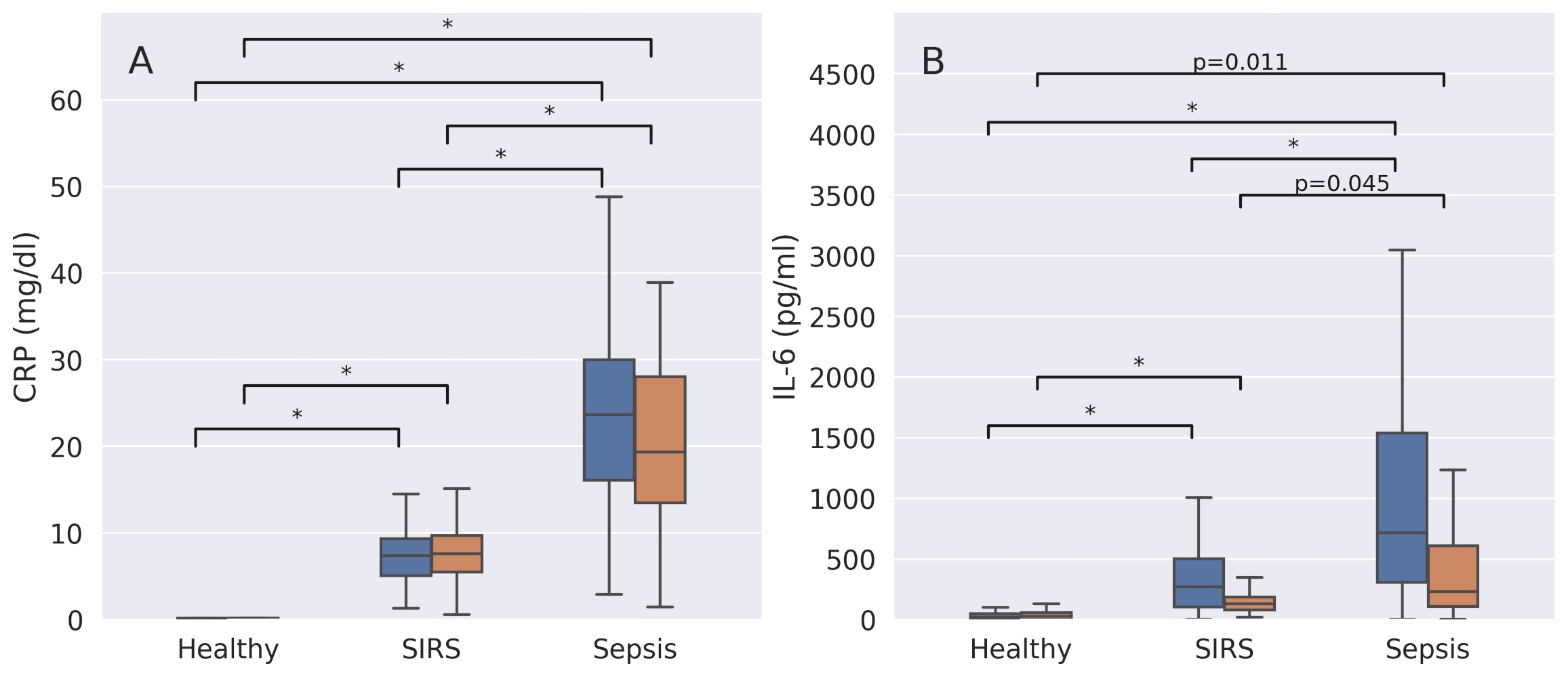


**Figure S3:** Comparison of C-reactive protein (CRP) (left graph A) and interleukin-6 (IL-6) (right graph B) plasma concentrations between healthy controls; patients with SIRS and septic patients. Data are presented separately for the exploratory (blue boxplots) and the confirmatory study (orange boxplots). All symbols indicate p<0.001; significant p-values ≥0.001 are given as numbers.


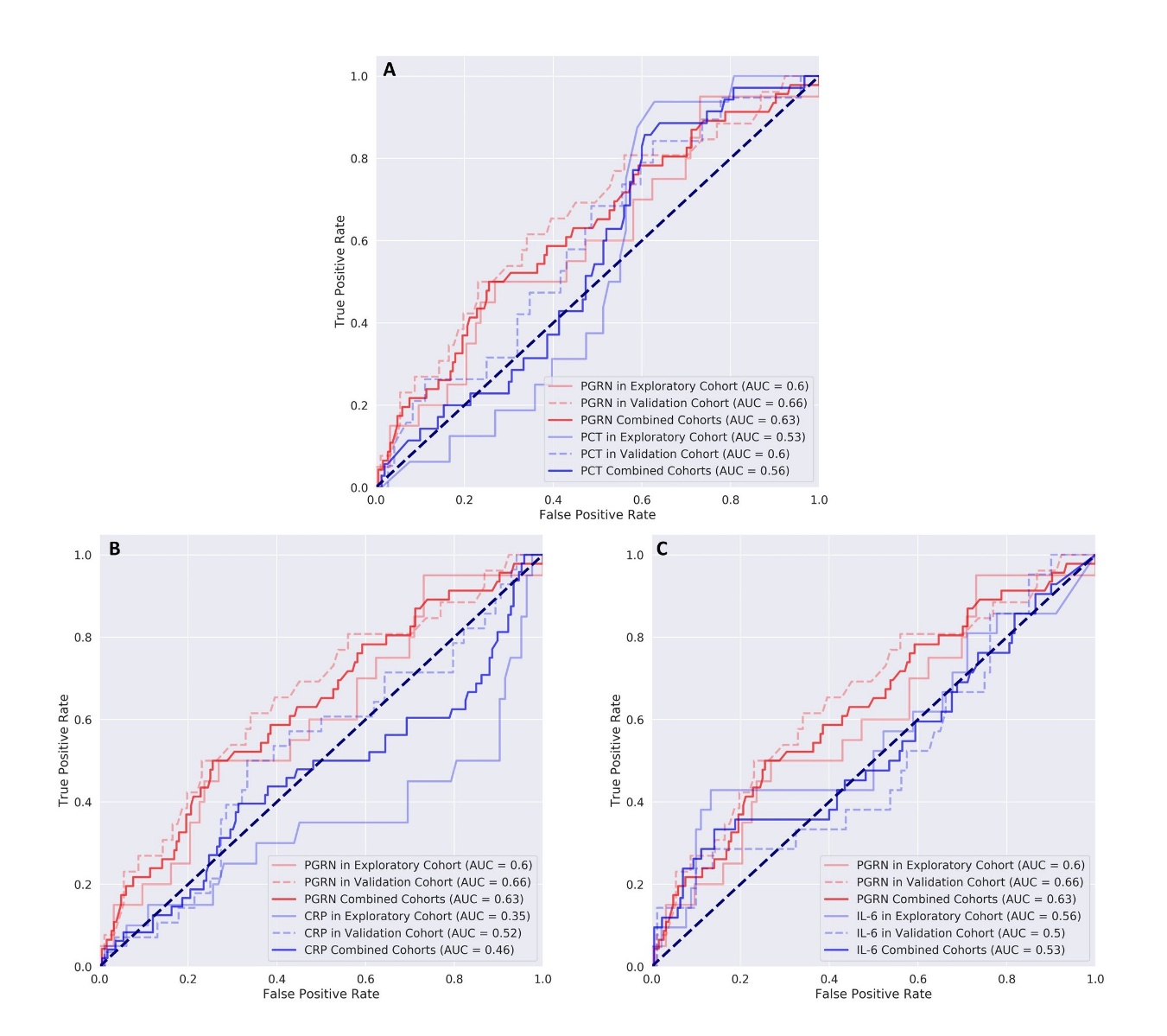


**Figure S4:** ROC curves and AUC values for mortality prediction between patients for (A) progranulin vs. procalcitonin; (B) progranulin vs. C-reactive protein; (C) progranulin vs. interleukin-6. The curves for progranulin are illustrated in red and the lines of the corresponding reference marker are outlined in blue; solid lines represent summary values from both cohorts. Lighter colors show measurements in the exploratory cohort and dashed lines the corresponding measurements in the confirmatory (validation) sample. AUC values and statistical comparison for progranulin and the reference biomarkers are presented in the main text and in Table 1 in the online supplement in more detail.


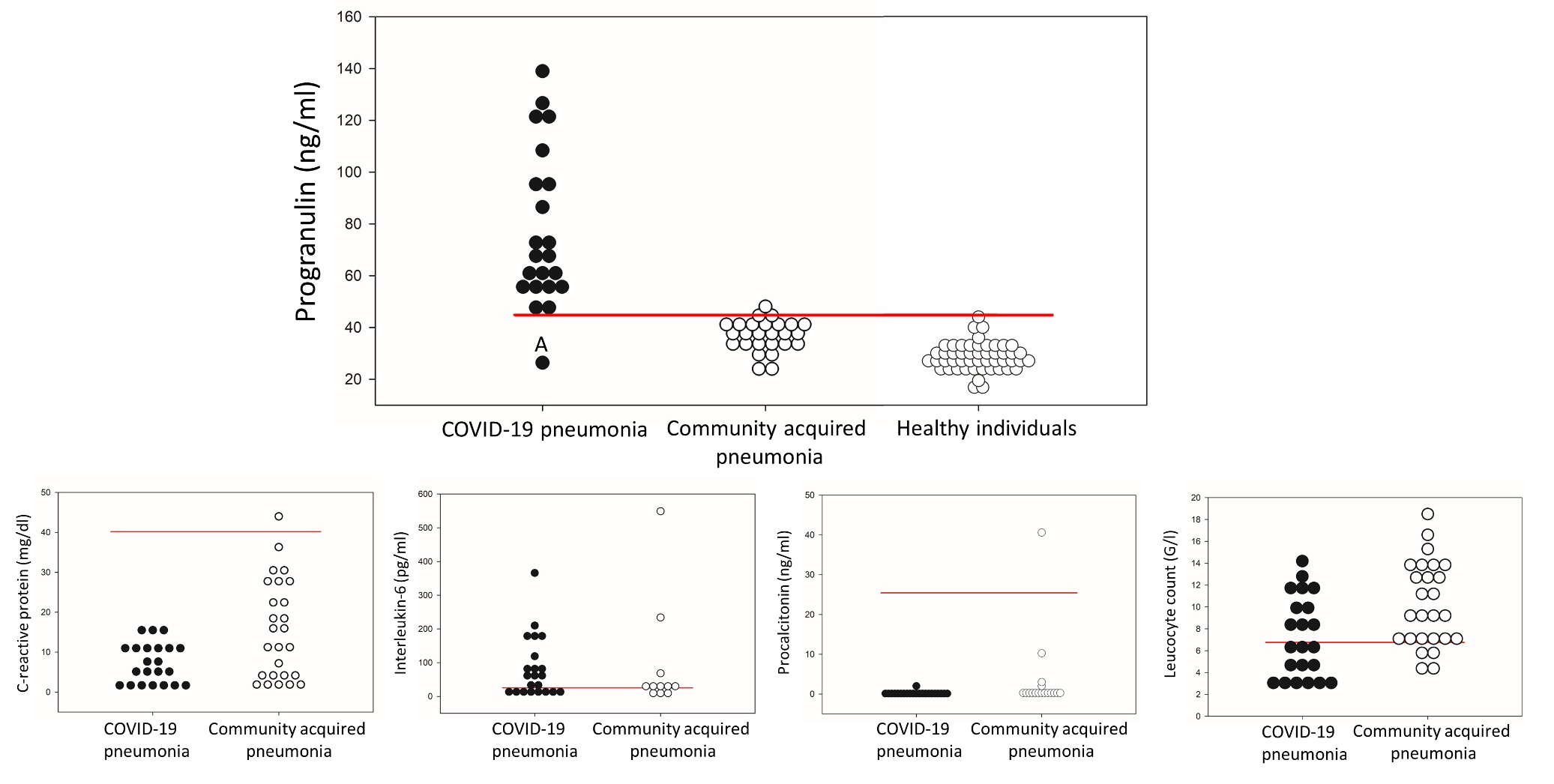


**Figure S5:** Dot histograms illustrating the diagnostic performance of progranulin for the differentiation between COVID-19 (n=22=, community acquired pneumonia (n=28) and healthy individuals (n=50). The lower row of graphs shows the absent diagnostic value of C-reactive protein, interleukin-6, procalcitonin and the leucocyte count in the study samples. Red lines indicate cut-off values. Outlier A in the progranulin histogram, presenting with lowest progranulin plasma concentrations in the healthy range (26.7 ng/ml) was mildly symptomatic for 14 days before hospital admission, was negatively tested for SARS-CoV-2 antibodies at admission and had only one positive nasal swap for SARS-CoV-19 at this time point; all three consecutive swaps were negative indicating low or no disease activity. A number of measurements were not available for interleukin-6 and procalcitonin.

| **Flow chart for patient inclusion** | | | | | | | | | | |
| --- | --- | --- | --- | --- | --- | --- | --- | --- | --- | --- |
|  |  |  |  |  |  |  |  |  |  |  |
| Sepsis |  |  |  | Localized infection |  |  |  | Pneumonia |  |  |
|  |  |  |  |  |  |  |  |  |  |  |
| n = 1186 |  |  |  | n = 86 |  |  |  | n = 97 |  |  |
|  |  |  |  |  |  |  |  |  |  |  |
|  | immunocompromized | n = 397 |  |  | immunocompromized | n = 16 |  |  | immunocompromized | n = 28 |
|  | no consent | n = 548 |  |  | no consent | n = 22 |  |  | no consent | n = 38 |
|  |  |  |  |  |  |  |  |  |  |  |
| n = 241 |  |  |  | n = 48 |  |  |  | n = 31 |  |  |
|  |  |  |  |  |  |  |  |  |  |  |

**Figure S6:** Flow chart representing the patient inclusion for sepsis, localized infection (infection) and community-acquired-pneumonia (pneumonia).
The patients included in the SIRS group were scheduled for elective open-heart surgery. Patients were included depending on the availability of the investigating study team

# References for online additional files

1. Shankar-Hari M, Phillips GS, Levy ML, Seymour CW, Liu VX, Deutschman CS, Angus DC, Rubenfeld GD, Singer M, Sepsis Definitions Task F, (2016) Developing a New Definition and Assessing New Clinical Criteria for Septic Shock: For the Third International Consensus Definitions for Sepsis and Septic Shock (Sepsis-3). JAMA 315: 775-787

2. Levy MM, Fink MP, Marshall JC, Abraham E, Angus D, Cook D, Cohen J, Opal SM, Vincent JL, Ramsay G, International Sepsis Definitions C, (2003) 2001 SCCM/ESICM/ACCP/ATS/SIS International Sepsis Definitions Conference. Intensive Care Med 29: 530-538

3. Bloos F, Trips E, Nierhaus A, Briegel J, Heyland DK, Jaschinski U, Moerer O, Weyland A, Marx G, Grundling M, Kluge S, Kaufmann I, Ott K, Quintel M, Jelschen F, Meybohm P, Rademacher S, Meier-Hellmann A, Utzolino S, Kaisers UX, Putensen C, Elke G, Ragaller M, Gerlach H, Ludewig K, Kiehntopf M, Bogatsch H, Engel C, Brunkhorst FM, Loeffler M, Reinhart K, for SepNet Critical Care Trials G, (2016) Effect of Sodium Selenite Administration and Procalcitonin-Guided Therapy on Mortality in Patients With Severe Sepsis or Septic Shock: A Randomized Clinical Trial. JAMA Intern Med 176: 1266-1276

4. Buschmann D, Kirchner B, Hermann S, Märte M, Wurmser C, Brandes F, Kotschote S, Bonin M, Steinlein OK, Pfaffl MW, Schelling G, Reithmair M, (2018) Evaluation of serum extracellular vesicle isolation methods for profiling miRNAs by next-generation sequencing. Journal of Extracellular Vesicles 7: 1481321

5. Hu J, Ge H, Newman M, Liu K, (2012) OSA: a fast and accurate alignment tool for RNA-Seq. Bioinformatics 28: 1933-1934

6. Love MI, Huber W, Anders S, (2014) Moderated estimation of fold change and dispersion for RNA-seq data with DESeq2. Genome Biol 15: 550

7. Vandesompele J, De Preter K, Pattyn F, Poppe B, Van Roy N, De Paepe A, Speleman F, (2002) Accurate normalization of real-time quantitative RT-PCR data by geometric averaging of multiple internal control genes. Genome Biol 3: RESEARCH0034

8. Andersen CL, Jensen JL, Orntoft TF, (2004) Normalization of real-time quantitative reverse transcription-PCR data: a model-based variance estimation approach to identify genes suited for normalization, applied to bladder and colon cancer data sets. Cancer Res 64: 5245-5250

9. Livak KJ, Schmittgen TD, (2001) Analysis of relative gene expression data using real-time quantitative PCR and the 2(-Delta Delta C(T)) Method. Methods 25: 402-408

10. Arechavaleta-Velasco F, Perez-Juarez CE, Gerton GL, Diaz-Cueto L, (2017) Progranulin and its biological effects in cancer. Med Oncol 34: 194

11. Jian J, Konopka J, Liu C, (2013) Insights into the role of progranulin in immunity, infection, and inflammation. J Leukoc Biol 93: 199-208

12. Rao L, Song Z, Yu X, Tu Q, He Y, Luo Y, Yin Y, Chen D, (2020) Progranulin as a novel biomarker in diagnosis of early-onset neonatal sepsis. Cytokine 128: 155000

13. Perkins NJ, Schisterman EF, (2005) The Youden Index and the optimal cut-point corrected for measurement error. Biom J 47: 428-441

14. DeLong ER, DeLong DM, Clarke-Pearson DL, (1988) Comparing the areas under two or more correlated receiver operating characteristic curves: a nonparametric approach. Biometrics 44: 837-845

15. Charlson ME, Pompei P, Ales KL, MacKenzie CR, (1987) A new method of classifying prognostic comorbidity in longitudinal studies: development and validation. J Chronic Dis 40: 373-383

16. Knaus WA, Draper EA, Wagner DP, Zimmerman JE, (1985) APACHE II: a severity of disease classification system. Crit Care Med 13: 818-829

17. Lopes JA, Jorge S, (2013) The RIFLE and AKIN classifications for acute kidney injury: a critical and comprehensive review. Clinical Kidney Journal 6: 8-14

18. Bone RC, (1993) A New simplified acute physiology score (SAPS II) based on a European/North American multicenter study. Journal of the American Medical Association 270: 2957-2963

19. Ferguson ND, Fan E, Camporota L, Antonelli M, Anzueto A, Beale R, Brochard L, Brower R, Esteban A, Gattinoni L, Rhodes A, Slutsky AS, Vincent JL, Rubenfeld GD, Thompson BT, Ranieri VM, (2012) The Berlin definition of ARDS: an expanded rationale, justification, and supplementary material. Intensive Care Med 38: 1573-1582

20. Youden WJ, (1950) Index for rating diagnostic tests. Cancer 3: 32-35
